# Precision-Driven Multi-Target Path Planning and Fine Position Error Estimation on a Dual-Movement-Mode Mobile Robot Using a Three-Parameter Error Model

**DOI:** 10.3390/s23010517

**Published:** 2023-01-03

**Authors:** Junjie Ji, Jing-Shan Zhao, Sergey Yurievich Misyurin, Daniel Martins

**Affiliations:** 1 Department of Mechanical Engineering, Tsinghua University, Beijing 100084, China; 2 Moscow Engineering Physics Institute, National Research Nuclear University MEPhI, Moscow 115409, Russia; 3 Blagonravov Mechanical Engineering Research Institute RAS, Malyi Kharitonievsky per.4, Moscow 101990, Russia; 4 Department of Mechanical Engineering, Federal University of Santa Catarina, Florianópolis 88040-900, Brazil

**Keywords:** mobile robots, fine error estimation, multi-point path planning

## Abstract

The multi-target path planning problem is a universal problem to mobile robots and mobile manipulators. The two movement modes of forward movement and rotation are universally implemented in integrated, commercially accessible mobile platforms used in logistics robots, construction robots, etc. Localization error in multi-target path tracking is one of the crucial measures in mobile robot applications. In this article, a precision-driven multi-target path planning is first proposed. According to the path’s odometry error evaluation function, the precision-optimized path can be discovered. Then, a three-parameter odometry error model is proposed based on the dual movement mode. The error model describes localization errors in terms of the theoretical motion command values issued to the mobile robot, the forward moving distances, and the rotation angles. It appears that the three error parameters follow the normal distribution. The error model is finally validated using a mobile robot prototype. The error parameters can be identified by analyzing the actual moving trajectory of arbitrary movements. The experimental localization error is compared to the simulated localization error in order to validate the proposed error model and the precision-driven path planning method. The OptiTrack motion capture device was used to capture the prototype mobile robot’s pose and position data.

## 1. Introduction

The target sequencing problem is a typical planning problem. One of the most famous target sequencing problems is the traveling salesman problem (TSP). It has been a long time since the TSP was first proposed. It is a problem of how to arrange the shortest possible route which visits each city once. There are many variants: the colored traveling salesman problem [1], the intermittent traveling salesman problem [2], the traveling salesman problem with release times [3], the traveling salesman problem with draft limits [4], and large-scale general colored traveling salesman [5]. As robotic technology advances, a mobile robot faces multi-target path planning problems in numerous circumstances [6,7,8,9]. The usual optimization criteria of the planning algorithm are the path length or the time cost [10,11,12]. There are other kinds of criteria for the path. Gao et al. [13] proposed a localizability evaluation method to find the path with optimal localizability. Zhang et al. [14] proposed criteria for considering the path length and angle and applied the path planning method in the greenhouse. However, the weights of the path length and path angle in the evaluation function in [14] are equal, which leads to a less meaningful application in reality.

Localization is one of the major tasks of mobile robot navigation. Wheel odometry is a critical foundation for localization in the sequencing of moving tasks. In the absence of an absolute positioning system, the positional error of mobile robots is accumulated. As the accumulated localization errors are different between different pathed sequences, a theoretically precise optimal path exists for the multi-point path planning problem. The prerequisite for solving the precision-driven multi-point path planning problem is the accurate error model for the mobile robot in sequencing its movements. Some motion error models for the mobile robot have been built. The error model of the mobile robot predicts position error in multiple-target tracking. The UMBmark [15] is a widely used, typical scheme of odometry error estimation based on the differential drive. The calibration strategy in UMBmark supposes that the wheel radius error and the wheelbase error are independent. Lee et al. [16] proposed an updated calibration strategy over UMBmark, considering that these two elements are affected by coupling. The disturbances of applied forces on the wheels are considered to derive the error model [17,18]. Filho et al. [19] proposed an analysis method for the impact of parametric uncertainties, in other words the non-systematic error parameters, on the localization error. However, these odometry error models strongly correspond to the structure of the mobile robot, such as the wheel radii, the distance between the wheels, the center of mass, etc. Most calibration methods are restricted to the limited specific structure of mobile robots.

Along with the development of robot technology, the complex integrated robot, for instance, the mobile manipulator, uses the integrated and commercial mobile base, such as the Segway RMP 440 Omni Flex [20], the HUSKY mobile platforms [21], etc. When facing a specific application for the mobile manipulator, the main program is usually considered the element motion with only two modes, moving forward and rotating in situ [22,23]. The requirement of the developers is the calibration functions on the moving distances and rotating angles in command without a detailed analysis of the structure’s properties of the mobile base. The odometry calibration strategy, which is responsible for the input commands to the mobile base, is required.

Based on the above analysis, this paper makes three major contributions to the current state of research.

First, a method for precision-driven multi-target path planning is proposed. The routing sequence of the known target positions expresses the resultant path. The evaluation function represents the localization precision of the path. In this study, the target positions are fixed and known. The precision-driven multi-target path planning problem in this study is also a target sequencing problem. As the genetic algorithm (GA) is valid for solving the traveling salesman problem [24,25] and target sequencing problems [26,27], the GA is used to find the optimal routing sequencing in this section.

Second, a three-parameter error model based on the dual-mode moving mobile robot is then proposed. The localization error of the path is expressed as a function of the input moving distances and the rotating angles. The proposed error model suits any mobile robot whose motion types are only moving forward and rotating in situ. The three parameters are the forward moving error coefficient, $k_{s}$, the rotation angle error coefficient, $k_{r}$, and the rotation center lateral offset, $d_{r}$. The properties and the rationalities of the proposed parameters are discussed in this article. Moreover, the compensation equations for the movement commands are proposed to increase the odometry precision based on the known error parameters.

Third, the error parameter identification method based on the random movements of the mobile robot is proposed. The error parameters are seen to follow the normal distribution. By collecting the moving trajectory of random movements and the corresponding moving commands of moving forward and rotating, the distribution of the model parameters can be calculated.

In this study, a prototype of a mobile robot was built to validate the formerly proposed contributions. Only the forward movement and rotation of the mobile robot are used to reach the target points. The motion capture system OptiTrack is commonly used to record dynamic motions [28,29]. The motion capture system records the detailed motion of all movements. To validate the proposed error model, comparisons were made between the simulated positions and the experimental positions in multiple-target tracking. Comparisons of both before and after motion calibration were made.

This paper is organized as follows. Section 2 proposes the formulation of the precision-driven multi-target path planning problem. The detail of the three parameters’ model is provided in Section 3. The parameters’ identification on the prototype mobile robot is shown in Section 4. The comparisons of the simulated stop positions and the experimental stop positions are reported in Section 5. The compensation methods based on the known error parameters to obtain a higher precision movement are introduced in Section 6. The discussion and conclusions are separately presented in Section 7 and Section 8.

## 2. Generalized Multiple-Target Path Planning Problem

**Figure 1:**
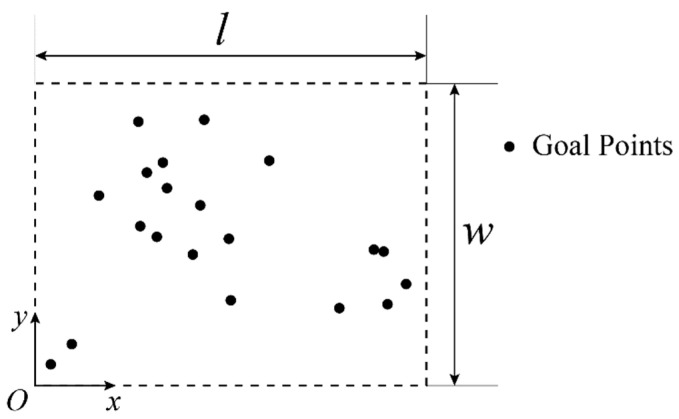
Workspace of the multiple-target path planning problem.

Suppose there is a workspace with the length of l and the width of w. There are n target positions scattered in this workspace. The target for the mobile robot is to move to each target point with the lowest costs. In Figure 1, the points in black denote the target positions. The path planning algorithms find the optimal path which connects each target position.

The set of target points can be expressed by

(1) $$G=\left\{g_{1},\cdot \cdot \cdot ,g_{n}\right\},g_{i}\in C$$

Each element $g_{i}$ represents one target position, and the number of the element in the set G is n, which denotes the number of target positions in the workspace.

As shown in Figure 1, a coordinate system is built on the ground of the workspace.

**Figure 2:**
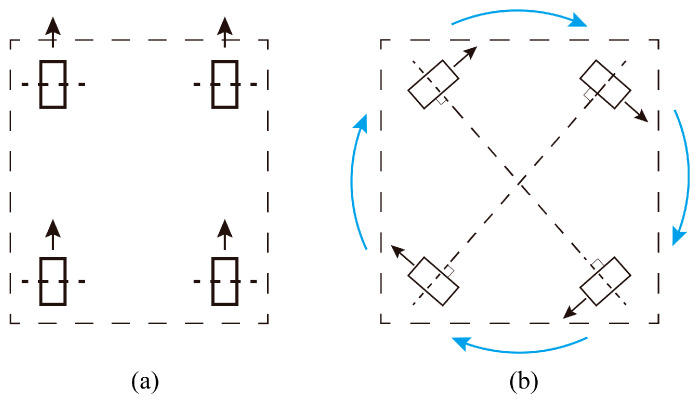
Structure examples for mobile and motion modes. (**a**) Moving forward. (**b**) Rotating in situ.

The motion modes for the robot in this article are forward movement and rotation in situ. The method of ideal rotation is to rotate across the center point of the mobile robot. Rotating around its center is a common ability for a mobile robot. As the example shows in Figure 2, a four-wheel robot has the capability of moving forward and rotating in situ. The arrows in black represent the motion direction of each wheel, and the arrows in blue represent the rotating direction around the center of the robot.

Figure 2a shows the mode of forward movement, and Figure 2b shows the mode of rotation in situ. In this situation, the total movements of the mobile robot moving along a multiple target points path can be composed of these two modes. The four-wheel structure shown in Figure 2 is just an example. The number of wheels can be two, three, or four. Even a mobile robot with crawlers suits the method proposed in this article.

Suppose that the initial position of the mobile robot is $P_{i}$; the next position the mobile robot moves to is $P_{i+1}$. The mobile robot first rotates in situ, and the forward direction of the mobile robot is adjusted towards the next position. Then, the mobile robot moves in the forward direction until reaching the target’s next position.

The quantitative values of these two motions are defined by the forward moving distances and turning angles moving from one target point to the next. However, the movement’s actual quantitative value is not the same as the expected quantitative value because of the movement error.

Because of this movement error, the actual positions are $P_{i}^{*}$, corresponding to the ideal positions, $P_{i}$. The subscript i denotes the indexes for the position. Thus, the average location error for the entire multi-target points path can be expressed.

(2) $$X=\frac{\sum_{i=1}^{n}d\left(P_{i},P_{i}^{*}\right)}{n}$$

The symbol X represents the average location error. The operator $d(P_{i},P_{i}^{*})$ represents the distance between the ideal position $P_{i}$ to the actual position $P_{i}^{*}$.

The motion error causes the position error at each movement, and the movements are classified into two modes, forward movement and rotation. Suppose that the moving distances of each forward movement is $l_{i}$, and the turning angle of each turning in situ is $\theta_{i}$. The motions can also express the average position error.

(3) $$X=f\left(L^{*},\theta^{*}\right)$$

Equation (3) represents the generalized evaluation function. The symbol $L^{*}$ in Equation (3) represents the actual distances array of moving forward.

(4) $$L^{*}=\left(l_{1}^{*},l_{2}^{*},\ldots ,l_{n}^{*}\right)$$

where the elements are the true distances, $l_{i}^{*}$, corresponding to the ideal distances, $l_{i}$.

The symbol $\theta^{*}$ represents the actual angles array of turning in situ.

(5) $$\theta^{*}=\left(\theta_{1}^{*},\theta_{2}^{*},\ldots ,\theta_{n}^{*}\right)$$

where the elements are the true angles, $\theta_{i}^{*}$, corresponding to the ideal angles, $\theta_{i}$.

The true motion vector, $\left(\theta_{i}^{*},l_{i}^{*}\right)$, can be seen as the function of the ideal motion pair, $\left(\theta_{i},l_{i}\right)$.

(6) $$[\theta_{i}^{*},l_{i}^{*}]=Ε\left(\theta_{i},l_{i}\right)$$

The structure of the function, $Ε\left(\theta_{i},l_{i}\right)$, is defined as the error function of the mobile robots. The error model is affected by the inner properties of the mobile robots.

According to Equations (3) and (5), as long as the structure of the evaluation function $f\left(L^{*},\theta^{*}\right)$ and $Ε\left(\theta_{i},l_{i}\right)$ are determined, the average position error can be seen as the optimization criterion to calculate the optimal multiple-target path.

The typical shortest total length path problem is a special status of Equation (3), as shown in Equation (7).

(7) $$X_{l}=f\left(L^{*},\theta^{*}\right)=\frac{\sum_{i=1}^{n}l_{i}}{n}$$

In Equation (7), the structure of the evaluation function is simplified to the summation of the distances between the adjacent target positions on the path.

**Figure 3:**
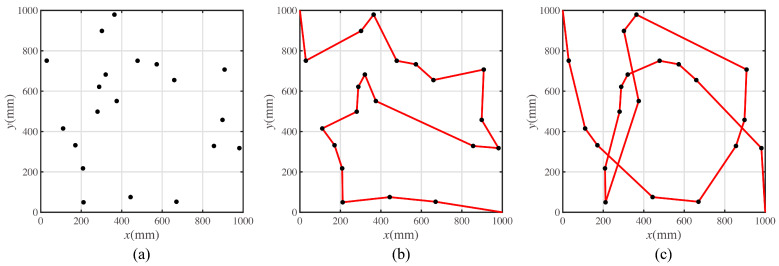
The target point distribution and the path solution. (**a**) Target points placed in the workspace. (**b**) The optimal path with the shortest length. (**c**) The optimal path with the least rotation.

Suppose that there are 20 target points placed in the square workspace. The coordinates of the target positions are shown in Table A1. The length of the workspace is 1000 mm, and the width of the workspace is 1000 mm, as shown in Figure 3a. The start point of the path is set as the top left point of the workspace, (0,1000), and the end point of the path is set as the bottom right point of the workspace, (1000,0).

The pattern in Figure 3b shows the shortest path result for the multi-target path planning problem.

What is more, when the summation of the rotation angle defines the evaluation function, the evaluation is shown in Equation (6).

(8) $$X_{l}=f\left(L^{*},\theta^{*}\right)=\frac{\sum_{i=1}^{n}\theta_{i}}{n}$$

Corresponding to the least summation of the turning angle, the optimal path is shown in Figure 3c.

In Figure 3, the dots in black represent the target points. The lines in red represent the moving paths for mobile robot. As the quantitative value of two characteristic movement modes are the moving distance and the rotating angle, the path shown in Figure 3a,b constitute the two characteristic paths. To recall these two characteristic paths simply, they are called the path with the shortest length and the path with the least rotation in the rest of this article.

## 3. Three Parameters’ Error Model for Mobile Robots

**Figure 4:**
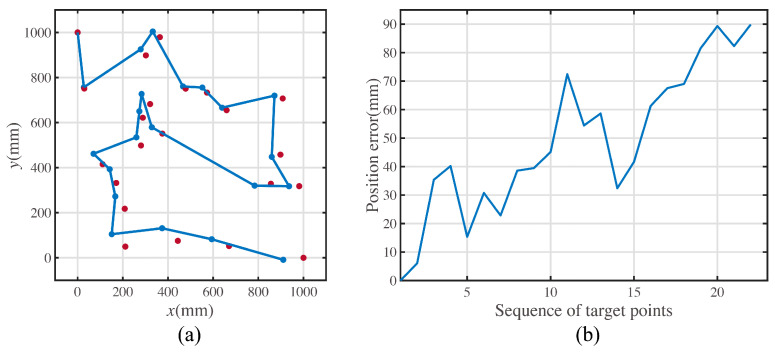
A realistic example of multiple-target position tracking. (**a**) Ideal positions and actual moving path in experiment. (**b**) Errors between the ideal positions and actual positions at each target point.

When the mobile robot executes the multiple-target path, the position the robot reaches is not the precise position of the target. Figure 4 shows realistic example experiment data for a mobile robot moving along the path with the shortest length.

Figure 4a shows the actual positions and the ideal positions. The points in red denote the ideal positions. In Figure 4a, the points and lines in blue denote the actual positions and moving paths captured by the motion capture system. Figure 4b shows the error polyline of the total movements. The error is defined as the distance between the actual and ideal positions.

The proposed error model is composed of three parameters, the forward moving error coefficient, $k_{s}$, the rotation angle error coefficient, $k_{r}$, and the rotation center lateral offset, $d_{r}$. In this part, the three parameters are explained in detail. Then, the position estimation equations of the multiple-target path are proposed.

### 3.1. Forward Moving Error Coefficient

Suppose that the expected moving distance is l, and the true moving distance is $l^{*}$. The proposed error model considers that the numerical relationship between the expected moving distance and the true moving distance is

(9) $$l^{*}=k_{s}l$$

In Equation (9), the coefficient of the expected moving distance, $k_{s}$, denotes the forward moving error coefficient, which is one of the three error parameters of the error model proposed in this article.

The forward moving error coefficient, $k_{s}$, can be seen as the comprehensive influence of the changing wheel radius and the changing tire slip.

The encoder, combined with the wheel’s axis, constitutes the feedback of the movement control. The controlled function of the wheel can be expressed by

(10) $$N=\frac{N_{0}l}{2\pi r}$$

In Equation (10), N denotes the pulse change in the wheel encoder, $N_{0}$ denotes the number of pulses per revolution of the encoder, r represents the radius of the wheel, and l denotes the moving distance to be controlled.

(11) $$l^{*}=2\pi rk_{s}\frac{N}{N_{0}}$$

Substituting Equation (9) into Equation (10), Equation (11) expresses the true control function for one wheel movement.

The forward moving distance error can be affected by the deformation of the transmission system, the deformation of the wheel, the tire slip, etc. These error factors can be summarized by the forward moving error coefficient, $k_{s}$.

### 3.2. Rotation Angle Error Coefficient

Suppose that the expected rotation angle is θ, and the true turning angle is $\theta^{*}$. The proposed error model considers that the numerical relationship between the expected rotation angle and the true turning angle is

(12) $$\theta^{*}=k_{r}\theta$$

In Equation (12), the coefficient of the expected turning angle, $k_{r}$, denotes the rotation angle error coefficient, which is one of the three error parameters of the error model proposed in this article.

The error in the rotation angle is also affected by the non-ideality of the mobile robot. Moreover, the contact situation on the tire between moving forward and turning is different. These two coefficients are seen as orthogonal.

### 3.3. Rotation Center Lateral Offset

Either in the forward moving error coefficient or the turning angle error coefficient, the moving speed of the wheel at each side of the mobile robot is considered the same. The difference in speed between the two sides of the mobile robot causes the rotation center lateral offset, $d_{r}$.

Because the speed of each side of the mobile robot is different, when the mobile robot rotates in situ, the true rotation center of the mobile robot shifts from the geometric center.

The rotation center lateral offset, $d_{r}$, is defined as the center offset projection on the direction which is normal to the forward direction of the mobile robot. Moreover, the center offset is defined as the segment which connects the true center and the geometric center.

Suppose there is an observer who stands at the geometric center of the mobile robot, and his/her forward direction is coincident with the forward direction of the mobile robot. If the true center is at the right of the observer, the numerical value of the rotation center lateral offset, $d_{r}$, is defined as positive. Similarly, if the true center is at the left of the observer, the numerical value of the rotation center lateral offset, $d_{r}$, is defined as negative.

The rotation center lateral offset reflects the wheel movement difference on two sides. No matter what the structure of the mobile robot is, two wheels, four wheels or other, the mobile robot can be seen as a symmetrical model. Figure 4 shows the geometrical relationship between the wheel movement difference and the rotation center lateral offset.

**Figure 5:**
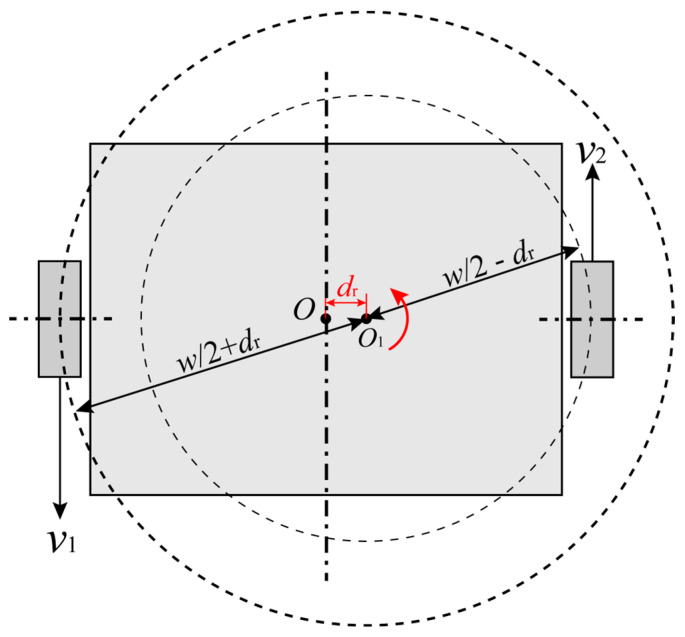
The rotation center lateral offset caused by the velocity difference.

As shown in Figure 5, the mobile robot is equivalently substituted by the two-wheel model. The left wheel moves backward with the velocity $v_{1}$. The right wheel moves forward with the velocity $v_{2}$. If $v_{1}=v_{2}$, the mobile robot rotates around the geometrical center. If $v_{1}\neq v_{2}$, the mobile robot rotates around the true center point, from which the distance to the geometrical center is $d_{r}$, which is shown in red in Figure 5. The arrow in red represents the rotation direction.

As the distances of the two wheels to the true center differ, the two wheels move along circularly with different radii. Suppose that the spacing between the wheels on two sides is w, then the radius of the left wheel trajectory is $w/2+d_{r}$, and the radius of the right wheel trajectory is $w/2-d_{r}$. The angular velocities of the two wheels are the same.

(13) $$\frac{v_{1}}{w/2+d_{r}}=\frac{v_{2}}{w/2-d_{r}}$$

Thus, the linear velocity ratio, $k_{w}$, of wheels on two sides is

(14) $$k_{w}=\frac{v_{1}}{v_{2}}=\frac{w+2d_{r}}{w-2d_{r}}$$

The offset of the rotation center affects the shape of the forward-moving trajectory. Because of the linear velocity difference between the two sides, the trajectory of moving forward performs like an arc, as shown in Figure 5.

**Figure 6:**
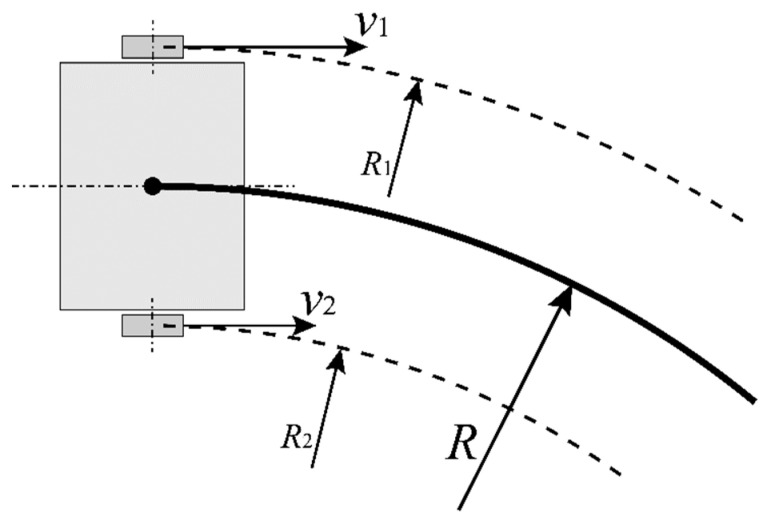
The arc-shaped trajectory caused by the rotation center lateral offset.

Figure 6 shows the true trajectory of moving forward. Suppose that the linear velocity of the left wheel is $v_{1}$, and the linear velocity of the right wheel is $v_{2}$. If $v_{1}=v_{2}$, the trajectory of moving forward is straight. If $v_{1}\neq v_{2}$, the trajectory of moving forward performs like an arc.

Suppose that the trajectory radius of the left wheel is $R_{1}$, and the trajectory radius of the right wheel is $R_{2}$. The linear velocity ratio between two wheels is also

(15) $$\frac{v_{1}}{v_{2}}=\frac{\omega R_{1}}{\omega R_{2}}=\frac{w+2d_{r}}{w-2d_{r}}$$

The angular velocities are the same at each point of the trajectory, and the trajectory radius of the true center of the mobile robot $R_{s}$ can be expressed by

(16) $$R_{s}=R_{1}-\frac{w}{2}=R_{2}+\frac{w}{2}$$

Substitute Equation (16) into Equation (15):

(17) $$R_{s}=\frac{w^{2}}{4d_{r}}$$

As the rotation center lateral offset, $d_{r}$, can be negative, the generalized trajectory radius of the true center can be negative in this situation.

According to Figure 5 and Figure 6, the trajectory caused by the offset of the rotation center and the non-linear trajectory of moving forward is the wheel linear velocity difference between the left and right equivalent wheels. Different factors lead to different slip rates between the tire and the ground.

Since the left and right sides of the general mobile robot often use the same type of motors and tires, the actual value of rotation center lateral offset is very small, far less than the left and right wheel spacing, so the impact on the rotation movement precision is also small.

Moreover, what the rotation center lateral offset, $d_{r}$, really affects is the trajectory shape of moving forward. The ideal straight motion of moving forward changes to the circular motion described by Equation (13). However, we define this effect as the rotation center lateral offset rather than the trajectory radius of moving forward because the true rotation center, compared with the circle center of the forward-moving trajectory, is on the mobile robot, not outside the mobile robot. Moreover, the rotation center lateral offset can be negative, corresponding to the circle center on the left.

The rotation center lateral offset parameter is used to explain the non-ideal property of moving forward, that is, the radius of the forward-moving trajectory $R_{s}$. Hence, only the nominal value of the wheel spacing, w, which is used in Equation (13), is needed.

The linear velocity difference between the wheels on both sides will affect the former two error parameters, $k_{s}$ and $k_{r}$, but this effect is so small that it can be ignored. This discussion will be presented in Section 4 with experimental data.

### 3.4. Error Evaluation Function in Multi-Target Path Moving

When the mobile robot moves along the multi-target path, the error at each position is related to each previous step. According to the known details of the error model at each step, the error performance of each point in the multi-target path can be estimated. At the same time, according to the error performance of each step, the motion path with the smallest error can be solved for the multi-objective path planning problem.

Firstly, the situation of a mobile robot moving between two points is discussed with the proposed three error parameters.

When the mobile robot executes the motion from the initial point, $P\left(x_{P},y_{P}\right)$, to the target point, $Q\left(x_{Q},y_{Q}\right)$, the mobile robot moves from the initial true point, $P^{\prime }\left(x_{P^{\prime }},y_{P^{\prime }}\right)$, to the true target point, $Q^{\prime }\left(x_{Q^{\prime }},y_{Q^{\prime }}\right)$. When the actual positions of the mobile robot are $P^{\prime }\left(x_{P^{\prime }},y_{P^{\prime }}\right)$ and $Q^{\prime }\left(x_{Q^{\prime }},y_{Q^{\prime }}\right)$, the robot thinks it is at positions $P\left(x_{P},y_{P}\right)$ and $Q\left(x_{Q},y_{Q}\right)$.

The true target position, $Q^{\prime }\left(x_{Q^{\prime }},y_{Q^{\prime }}\right)$, can be expressed by

(18) $$\left[\begin{array}{c} x_{Q^{\prime }}\\ y_{Q^{\prime }} \end{array}\right]=\left[\begin{array}{c} x_{P^{\prime }}\\ y_{P^{\prime }} \end{array}\right]+\left[\begin{array}{cc} \cos\left(\frac{2d_{r}k_{s}d_{PQ}}{w^{2}}\right) & \sin\left(\frac{2d_{r}k_{s}d_{PQ}}{w^{2}}\right)\\ -\sin\left(\frac{2d_{r}k_{s}d_{PQ}}{w^{2}}\right) & \cos\left(\frac{2d_{r}k_{s}d_{PQ}}{w^{2}}\right) \end{array}\right]\left[\begin{array}{c} k_{s}\left(x_{Q}-x_{P}\right)\\ k_{s}\left(y_{Q}-y_{P}\right) \end{array}\right]$$

In Equation (18), $d_{PQ}$ denotes the distance from point P to Q, $k_{s}$ denotes the forward moving error coefficient, $d_{r}$ denotes the rotation center lateral offset, and w denotes the nominal spacing between the wheels on both sides. The moving error between two points is independent of the turning angle error coefficient.

If the motion from point $P\left(x_{P},y_{P}\right)$ to point $Q\left(x_{Q},y_{Q}\right)$ is considered ideal, then the final pose vector after the movement is

(19) $$p_{Q}=\left[\begin{array}{c} x_{Q}-x_{P}\\ y_{Q}-y_{P} \end{array}\right]$$

However, the movement is non-ideal. The true pose vector is

(20) $$p_{Q^{\prime }}=\left[\begin{array}{c} x_{Q^{\prime }}-\frac{x_{Q}+2x_{P^{\prime }}-x_{P}}{2}\\ y_{Q^{\prime }}-\frac{y_{Q}+2y_{P^{\prime }}-y_{P}}{2} \end{array}\right]$$

Equation (20) is an approximate equation. The approximate condition is

(21) $$\left|R_{s}\right|=\left|w^{2}/4d_{r}\right|\gg d_{PQ}$$

If the approximate condition is invalid, the true pose vector is

(22) $$p_{Q^{\prime }}=\left[\begin{array}{c} x_{Q^{\prime }}-x_{P^{\prime }}-R_{s}\left(\frac{x_{Q}-x_{P}}{d_{PQ}}\right)\tan\left(\frac{d_{PQ}}{2R_{s}}\right)\\ y_{Q^{\prime }}-y_{P^{\prime }}-R_{s}\left(\frac{y_{Q}-y_{P}}{d_{PQ}}\right)\tan\left(\frac{d_{PQ}}{2R_{s}}\right) \end{array}\right]$$

The movement error is irrelevant to the turning angle error coefficient in this situation.

Next, the error performance of continuous movements between multiple points is discussed.

The movement discussed in Equation (14) supposes that the initial pose is from the ideal initial point P to the ideal target point Q. That is,

(23) $$p_{0}=p_{Q}=\left[\begin{array}{c} x_{Q}-x_{P}\\ y_{Q}-y_{P} \end{array}\right]$$

However, before the mobile robot executes the movement from the ideal initial point P to the ideal target point Q, the ideal initial pose $p_{P}$ is dependent on the former movement before reaching point P. The initial true pose at point $P^{\prime }$ is $p_{P^{\prime }}$, which is dependent on every former movement.

Hence, the actual movements between the true point $P^{\prime }\left(x_{P^{\prime }},y_{P^{\prime }}\right)$ and the true point $Q^{\prime }\left(x_{Q^{\prime }},y_{Q^{\prime }}\right)$ can be expressed as below. Firstly, the mobile robot turns in situ. The forward direction is adjusted to the next target point. Then, the mobile robot moves forward straightly to the next target point. The turning angle error coefficient affects the actual turning angle in the rotation movement. The movements are shown in Figure 6.

**Figure 7:**
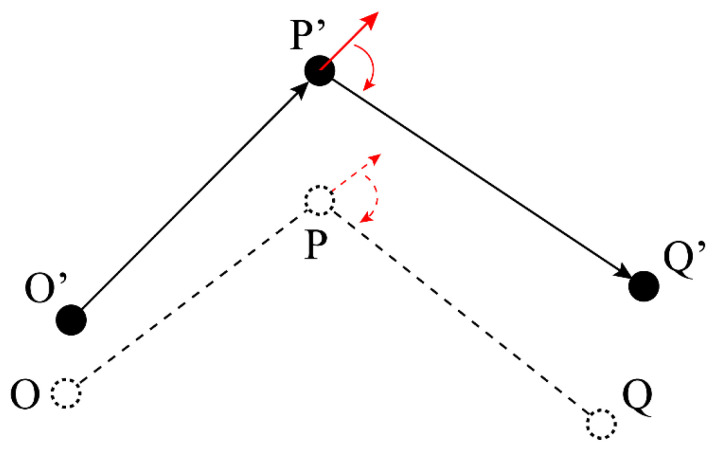
Position error when moving from one target point to the next one.

As shown in Figure 7, when the mobile robot finishes the movement from point O to point P, the ideal position is $P\left(x_{P},y_{P}\right)$, and the true position is $P^{\prime }\left(x_{P^{\prime }},y_{P^{\prime }}\right)$. The ideal pose vector is $p_{P}={\left(x_{P}-x_{O},y_{P}-y_{O}\right)}^{T}$. The true initial pose vector is $p_{P^{\prime }}={\left(x_{P^{\prime }}-x_{O^{\prime }},y_{P^{\prime }}-y_{O^{\prime }}\right)}^{T}$.

In Figure 7, the straight arrows in red represent the forward direction when the robot reach the points P and $P^{\prime }$. And the curved arrows in red represent the rotation movements before moving to the points Q and $Q^{\prime }$. Before the mobile robot moves from point $P^{\prime }$ to point $Q^{\prime }$, the mobile robot turns in situ. The ideal turning angle $\theta_{P}$ is

(24) $$\theta_{P}=\arccos\frac{p_{P}\cdot p_{Q}}{{\left\|p_{P}\right\|}_{2}{\left\|p_{Q}\right\|}_{2}}$$

Because of the turning angle error coefficient, $k_{r}$, the true turning angle is

(25) $$\theta_{P^{\prime }}=k_{r}\theta_{P}$$

Hence, the value difference between the ideal turning angle and the true turning angle is

(26) $$\theta_{P^{\prime }}-\theta_{P}=\left(k_{r}-1\right)\theta_{P}$$

Then, the revised equation for Equation (18) is

(27) $$\left[\begin{array}{c} x_{Q^{\prime }}\\ y_{Q^{\prime }} \end{array}\right]=\left[\begin{array}{c} x_{P^{\prime }}\\ y_{P^{\prime }} \end{array}\right]+\left[\begin{array}{cc} \cos\left(\beta \right) & \sin\left(\beta \right)\\ -\sin\left(\beta \right) & \cos\left(\beta \right) \end{array}\right]\left[\begin{array}{c} k_{s}\left(x_{Q}-x_{P}\right)\\ k_{s}\left(y_{Q}-y_{P}\right) \end{array}\right]$$

In Equation (27), β represents the equivalent turning angle:

(28) $$\beta =\frac{2d_{r}k_{s}d_{PQ}}{w^{2}}+\left(k_{r}-1\right)\theta_{P}$$

According to Equation (22), when considering the turning angle error, the final pose after reaching the target point, $p_{Q^{\prime }}$, is

(29) $$p_{Q^{\prime }}=\left[\begin{array}{c} x_{Q^{\prime }}-R_{s}\left(\frac{x_{Q}-x_{P}}{l_{PQ}}\right)\tan\left(\frac{d_{PQ}}{2R_{s}}\right)\\ y_{Q^{\prime }}-R_{s}\left(\frac{y_{Q}-y_{P}}{d_{PQ}}\right)\tan\left(\frac{d_{PQ}}{2R_{s}}\right) \end{array}\right]-\left[\begin{array}{cc} \cos\left(k_{r}\theta_{P}\right) & \sin\left(k_{r}\theta_{P}\right)\\ -\sin\left(k_{r}\theta_{P}\right) & \cos\left(k_{r}\theta_{P}\right) \end{array}\right]p_{P^{\prime }}$$

If the approximate condition (21) is valid, then the approximate equation of Equation (29) is

(30) $$p_{Q^{\prime }}=\left[\begin{array}{c} x_{Q^{\prime }}-\frac{x_{Q}-x_{P}}{2}\\ y_{Q^{\prime }}-\frac{y_{Q}-y_{P}}{2} \end{array}\right]-\left[\begin{array}{cc} \cos\left(k_{r}\theta_{P}\right) & \sin\left(k_{r}\theta_{P}\right)\\ -\sin\left(k_{r}\theta_{P}\right) & \cos\left(k_{r}\theta_{P}\right) \end{array}\right]p_{P^{\prime }}$$

For the multi-target path planning problem, the set of target points is known. Using the coordinate point iteration Equation (27) and the pose vector iteration Equation (29), the actual positions from the start to the end can be estimated. The position error of any path can be estimated.

## 4. Error Parameters’ Estimation on Prototype Experiment

A mobile robot prototype was built, and the motion capture system was used to capture the movement of the mobile robot. The numerical value of the error parameters was estimated from the prototype experiment.

### 4.1. Error Evaluation Function in Multi-Target Path Moving

**Figure 8:**
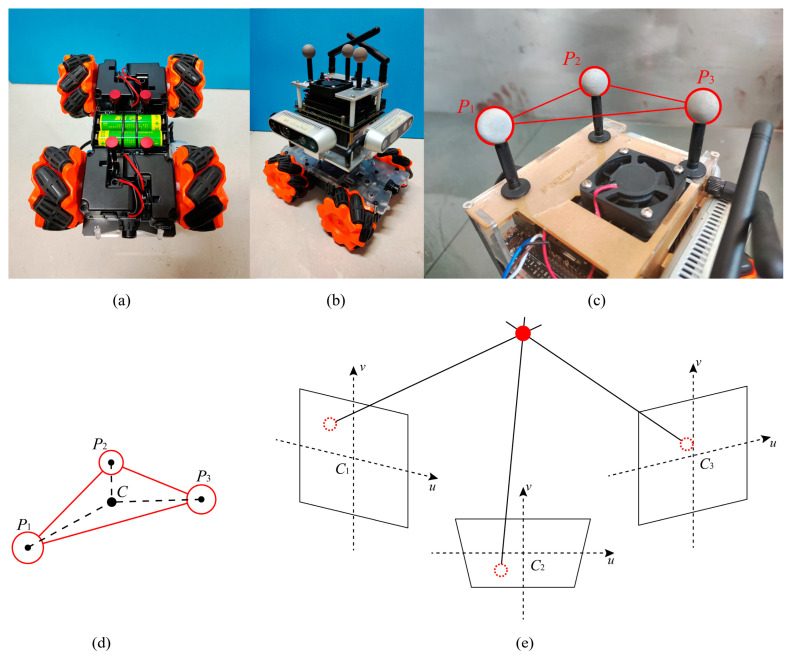
Prototype of mobile robot and the motion capture system. (**a**) The four Mecanum wheels of the mobile robot. (**b**) The overall view of the mobile robot prototype. (**c**) Marked points on the mobile robot. (**d**) The rigid triangle formed by marked points that represents the motion of the mobile robot. (**e**) The geometric principle of the multiple-camera stereo system.

The prototype was composed of the chassis with four Mecanum wheels, as shown in Figure 8a,b. The Mecanum wheels were arranged symmetrically to implement the function of moving forward and rotating in situ. Figure 8b shows the overall view of the mobile robot.

The motion capture system uses the principle of a multi-view stereo vision system to calculate the 3-D coordinates of the marked points. Four motion capture cameras (Prime 17W, OptiTrack) were arranged to capture the sequenced coordinates of the marked points.

Three marked points were fixed to the top of the mobile robot, as shown in red in Figure 8c. The three points were arranged at the same height. The motion of the triangle center, C, represents the motion of the mobile robot, as shown in Figure 8d. The mobile robot moved on the ground. The coordinates of point C projected on the ground represent the coordinates of the mobile robot. The pose of the triangle, $P_{1}P_{2}P_{3}$, represents the pose of the mobile robot.

The multiple-camera stereo system of OptiTrack is shown in Figure 8e. A marked point was observed by multiple cameras. The hollow circles in red represents the images of the marked points on the image coordinate system of each camera. The solid circle in red represents the calculated 3-D coordinates of the marked points according to the geometric relationship between cameras. The precision of the captured data depended on the number of cameras and the arranged geometry relationships. The error in the captured coordinates was smaller than 1 mm.

### 4.2. High-Precision Motion Recognition with Motion Capture System

The problem for motion recognition from time series data generated by the motion capture system is distinguishing between the motion and stationary segments. The vibration and disturbance of marked points constitute the noise in recorded 3-D coordinate data. According to the proposed three-parameter error model, the forward moving error coefficient, $k_{s}$, corresponds to the distance difference between the expected moving distance and the actual moving distance. The turning angle error coefficient, $k_{r}$, corresponds to the difference between the expected rotating angle and the actual rotating angle. The rotation center lateral offset, $d_{r}$, corresponds to the shape of a forward-moving trajectory.

**Figure 9:**
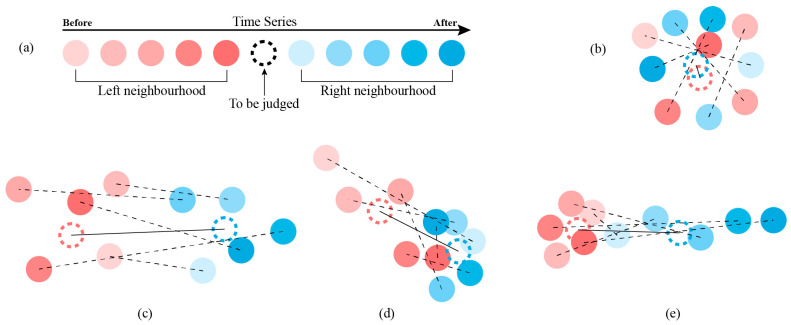
The algorithm principle of stationary segment recognition. (**a**) The coordinates of the same marked point in time series. (**b**) The coordinate series in stationary status. (**c**) The coordinate series in motive status. (**d**) The coordinate series when the marked point is about to stop. (**e**) The coordinate series when the marked point is about to move.

The actual moving distance and actual rotating angle, which correspond to the parameters $k_{s}$ and $k_{r}$, are calculated from the stationary status of the mobile robot before and after movement. The arc-shaped trajectory is fit from the forward moving point series. Thus, a precise algorithm to distinguish between the stationary and motion segments is required to recognize the error parameters. Figure 9 shows the principle diagram of the proposed algorithm.

As shown in Figure 9a, the data generated from the motion capture system comprise a time coordinate series of a specific marked point. The coordinates series just before the point to be distinguished is called the left neighborhood. The coordinates series just after the point to be distinguished is called the right neighborhood. The left neighborhood positions are marked in different reds. The right neighborhood positions are marked in different blues.

Figure 9b shows an example of the stationary segment. The dotted unfilled circles in red and blue represent the average coordinates of the left and right neighborhoods. If the middle point to be judged is stationary, the coordinates of the left and right neighborhoods should be close.

Figure 9c shows the motion status. The distance between the two average coordinates is significant. If the middle point to be judged is motion, the average coordinates of the left neighborhood and the average coordinates of the right neighborhood are far from each other. The velocity of the middle point affects the numerical value of the distance.

Suppose that the point coordinates at the time t to be distinguished are

(31) $$p_{t}={\left(x_{t},y_{t},z_{t}\right)}^{T}$$

The left neighborhood matrix, $S_{t,l}$, can be expressed as

(32) $$S_{t,l}=\left[\begin{array}{ccc} p_{t-m} & \ldots & p_{t-1} \end{array}\right]$$

The right neighborhood matrix, $S_{t,r}$, can be expressed as

(33) $$S_{t,r}=\left[\begin{array}{ccc} p_{t+1} & \ldots & p_{t+m} \end{array}\right]$$

where the integer m denotes the length of the neighborhood.

The coordinates of a specific point can be seen as a vector with three elements. A difference vector between two neighborhoods is defined to standardize the distinguishing criterion.

The difference vector of the neighborhood, ${\tilde{s}}_{t}$, is defined as

(34) $${\tilde{s}}_{t}=\left(D\left(p_{t-m},p_{t+1}\right),\ldots ,D\left(p_{t-1},p_{t+m}\right)\right)=\left(d_{1},\ldots ,d_{m}\right)$$

where the operator $D\left(p_{i},p_{j}\right)$ represents the function of calculating the distance between point $p_{i}$ and point $p_{j}$. The element $d_{i}$ represents the distances of the points from the point of the left neighborhood to the corresponding point of the right neighborhood.

The average difference, $\bar{d}$, is defined as

(35) $$\bar{d}=\frac{\sum_{i=1}^{m}d_{i}}{m}$$

The variance of differences, $\sigma_{d}^{2}$, is defined as

(36) $$\sigma_{d}^{2}=\frac{\sum_{i=1}^{m}{\left(d_{i}-\bar{d}\right)}^{2}}{m-1}$$

Only when the average difference $\bar{d}$ and variance of differences, $\sigma_{d}^{2}$, are smaller than a specific threshold, $d_{\max}$ and $\sigma_{\max}^{2}$, can the middle point to be distinguished be called a stationary point. The value of the threshold depends on the noisy degree of the motion capture system.

Figure 9d,e show the status when the middle point is going to stop and going to start moving. The distance between two average coordinates is smaller. However, the variance of differences is larger compared with the pattern shown in Figure 9c.

**Figure 10:**
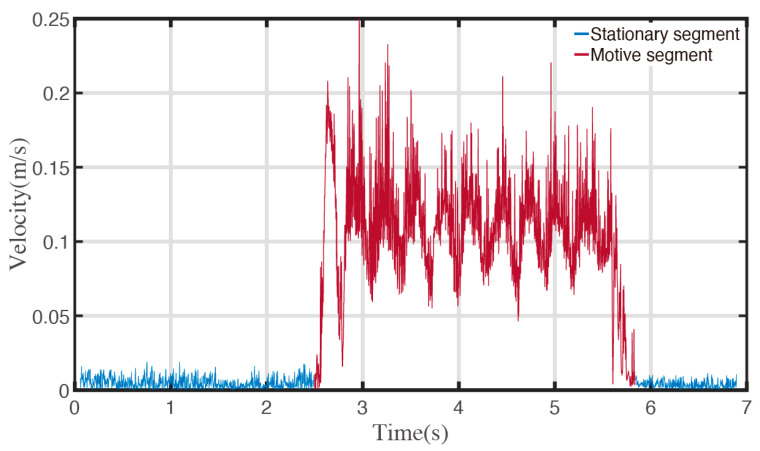
Stationary segment recognition.

Figure 10 shows an example of the stationary segmentation of continuous coordinate data captured by the motion capture system.

As shown in Figure 10, to express the difference between the motive segment and the stationary segment better, the captured data are shown with the velocity sequences. The velocity at a specific time is defined as the quotient of the distance between adjacent points by the time change in these two captured points. The motion segment is shown in red and the stationary segment is shown in blue.

Using the proposed stationary segment distinguishing algorithm, the entire motion capture coordinate series can be processed to the motion series, composed of the distances of moving forward and angles of rotating in situ.

### 4.3. Parameter Recognition Based on Arbitrary Mobile Robot Motions

To better recognize the error parameters, the movement should simulate the multi-target moving scenarios. Repeating the motion of moving forward or rotating in situ to generate the true data of the motion does not reflect the real situation.

**Figure A1:**
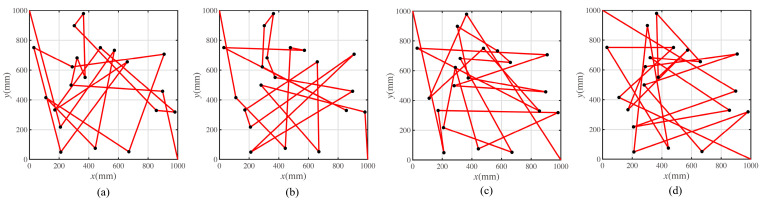
Random generated paths for parameter recognition. (**a**) The first random moving path. (**b**) The second random moving path. (**c**) The third random moving path. (**d**) The fourth random moving path.

Four random paths for the point pattern of Figure 3a were generated to act as the motion dataset, as shown in Figure A1.

Every expected value of movement and the true value of movement were calculated to fit the value of the error parameters.

**Figure 11:**
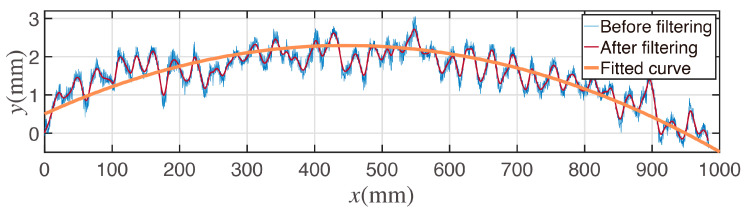
Arc fitting for forward-moving trajectory.

An arc-shaped forward-moving trajectory segment is shown in Figure 11; the trajectory shape of the forward movement is obviously arc-shaped. The line in blue represents the initial data of the motion capturing. The line in red represents the center trajectory after the filter. The line in orange represents the fitted arc segment.

According to the forward-moving trajectory shown in Figure 11, the value of the rotation center lateral offset is positive.

By extracting every segment of the forward movement, the rotation center lateral offset can be recognized. The original data of the expected forward moving distance and actual forward moving distance are shown in Table A2. Moreover, the original data of expected and actual rotation angles are shown in Table A3.

**Figure 12:**
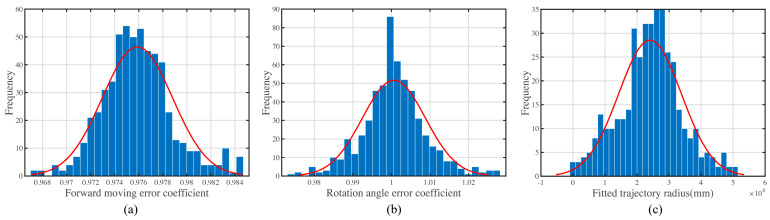
The distribution of the error parameter from experiments. (**a**) The distribution of the forward moving error coefficient. (**b**) The distribution of the rotation angle error coefficient. (**c**) The distribution of the fitted trajectory radius.

Firstly, the forward moving error coefficient was fit with the ratio of the true distance and the expected moving distances, as shown in Figure 12a. In the three subfigures of Figure 12, the columns in blue represent the frequency at each value interval. The curve in red represents the fitted normal distribution function curve.

Secondly, the turning angle error coefficient was fit with the ratio of the true angles and the expected angles, as shown in Figure 12b.

The trajectory for forward-moving trajectory is arc-shaped. The radius of the trajectories was fitted for the error parameter recognition. Finally, the rotation center lateral offset fits with the arc fitting of the forward-moving trajectory, as shown in Figure 12c.

The result of the distribution fitting shows a normal distribution. The parameters of the normal distributions are shown in Table 1. The rotation center lateral offset, $d_{r}$ is calculated according to Equation (17), where the wheel track is w=120 mm.

As shown in Table 1, the true rotation angle is usually larger than the expected rotation angle. In contrast, the actual forward moving distance is usually smaller than the expected forward moving distance.

The variance in $k_{r}$ is much larger than the variance in $k_{s}$. After the error parameters are known, the experiment validates the error model. The error model was made to forecast the distribution of the multi-target moving error for a mobile robot.

### 4.4. Rationality Discussion of Error Parameters

**Figure 13:**
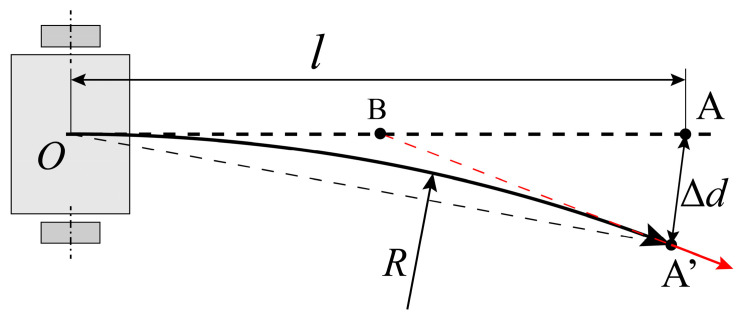
Destination offset caused by arc-shaped trajectory.

According to the fitted data shown in Table 1, the rationality of the proposed three error parameters model can be proved. Substitute the fitted trajectory radius R into Equation (17). The rotation center lateral offset, $d_{r}$, is negative. Hence, when the mobile robot prototype moves forward, the actual target position is always located at the ideal destination’s right, as shown in Figure 13.

Suppose that the ideal forward moving distance for the mobile robot is l. Because of the error parameter, $d_{r}$, the actual shape of the trajectory is an arc. The radius of the trajectory is $R_{s}$, and the arc length is also l. The ideal destination is point A, and the actual position after moving is point $A^{\prime }$. The arrow in red represents the forward direction of the mobile robot when it reach the point $A^{\prime }$. The starting point is O. The central angle of this circular movement is

(37) $$\alpha =\frac{l}{R_{s}}$$

The corresponding chord length of the trajectory is

(38) $$d_{OA^{\prime }}=2R_{s}\sin\frac{\alpha }{2}$$

Substituting Equation (37) into Equation (38), the ratio of the chord length to the arc length is

(39) $$\frac{d_{OA^{\prime }}}{l}=2\frac{R_{s}}{l}\sin\frac{l}{2R_{s}}$$

Substituting the value of the error parameters and ideal length, l=1000 mm, into Equation (39), the value of the ratio is $d_{OA^{\prime }}/l=0.999928$. Moreover, the difference between the chord length and the arc length is $d_{OA^{\prime }}-l=0.072\;\mathrm{mm}$. However, according to the value of the forward moving error coefficient, the actual forward moving error is about 25 mm. The error caused by the error parameter, $k_{s}$, is much larger than the error caused by the error parameter, $d_{r}$. If the trajectory radius is much larger than the moving distance, R≫l, the trajectory displacement can be seen as equal to the trajectory length, $d_{OA^{\prime }}\approx l$. Therefore, the forward moving error coefficient, $k_{s}$, can be seen as orthogonal to the rotation center lateral offset, $d_{r}$.

However, the distance between the expected and actual target positions cannot be ignored. The triangle $△AOA^{\prime }$ can be seen as an isosceles triangle. The vertex angle, β, of this isosceles triangle is

(40) $$\beta =\frac{\alpha }{2}$$

where α denotes the central angle shown in Equation (37). The distance between the target position and the actual ending position, Δd, can be expressed as

(41) $$\Delta d=2l\sin\frac{\beta }{2}$$

Substitute Equations (37) and (40) into Equation (41):

(42) $$\Delta d=2l\sin\frac{l}{4R_{s}}$$

Then, substitute the prototype parameters and the ideal forward moving distance l=1000 m into Equation (42): Δd=20.78 mm. This distance error is significant enough.

Not only is the error of the destination offset significant, the error between the ideal posture and the actual posture is also significant.

**Figure 14:**
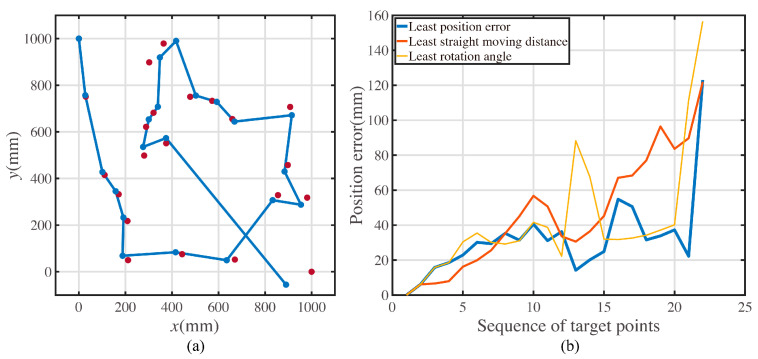
Precision optimal path and the comparison with the characteristic path. (**a**)The ideal positions and the actual reached positions when the mobile robot moves along the precision-driven optimal path. (**b**)The distances between the ideal positions and the actual reached positions at each target position when mobile robot moves along three different paths.

As shown in Figure 14, suppose that the starting direction vector is $p_{start}=\vec{OA}$, and the ending direction vector is $p_{end}=\vec{BA^{\prime }}$. The starting coordinate is $O\left(x_{O},y_{O}\right)$, and the ideal destination coordinate is $A\left(x_{A},y_{A}\right)$. The actual ending position coordinate is $A^{\prime }\left(x_{A^{\prime }},y_{A^{\prime }}\right)$.

According to the discussed geometrical relationship shown in Figure 14, the movement from point $O\left(x_{O},y_{O}\right)$ to point $A^{\prime }\left(x_{A^{\prime }},y_{A^{\prime }}\right)$ can be expressed by a displacement and a rotation. The coordinate transformation equation shown in Equation (43) expresses the movement from point $O\left(x_{O},y_{O}\right)$ to point $A^{\prime }\left(x_{A^{\prime }},y_{A^{\prime }}\right)$.

(43) $$\left[\begin{array}{c} x_{A^{\prime }}\\ y_{A^{\prime }} \end{array}\right]=\left[\begin{array}{c} x_{O}\\ y_{O} \end{array}\right]+\left[\begin{array}{cc} \cos\left(\beta \right) & \sin\left(\beta \right)\\ -\sin\left(\beta \right) & \cos\left(\beta \right) \end{array}\right]\left[\begin{array}{c} x_{A}-x_{O}\\ y_{A}-y_{O} \end{array}\right]$$

where β represents the vertex angle of the isosceles triangle shown in Equation (40). Substitute Equations (17) and (37) into Equation (40):

(44) $$\beta =\frac{2d_{r}l}{w^{2}}$$

According to the geometrical properties of the circular motion, the coordinates of point $B\left(x_{B},y_{B}\right)$ are

(45) $$\left[\begin{array}{c} x_{B}\\ y_{B} \end{array}\right]=\left[\begin{array}{c} x_{O}+R_{s}\left(\frac{x_{A}-x_{O}}{l}\right)\tan\left(\frac{l}{2R_{s}}\right)\\ y_{O}+R_{s}\left(\frac{y_{A}-y_{O}}{l}\right)\tan\left(\frac{l}{2R_{s}}\right) \end{array}\right]$$

Thus, the ending direction vector $p_{end}$ can be expressed as

(46) $$p=\left[\begin{array}{c} x_{A^{\prime }}-x_{B}\\ y_{A^{\prime }}-y_{B} \end{array}\right]=\left[\begin{array}{c} x_{A^{\prime }}-x_{O}-R_{s}\left(\frac{x_{A}-x_{O}}{l}\right)\tan\left(\frac{l}{2R_{s}}\right)\\ y_{A^{\prime }}-y_{O}-R_{s}\left(\frac{y_{A}-y_{O}}{l}\right)\tan\left(\frac{l}{2R_{s}}\right) \end{array}\right]$$

If the approximate condition, $R_{s}=\left|w^{2}/4d_{r}\right|\gg l$, is valid, Equation (46) can be approximated as

(47) $$p=\left[\begin{array}{c} x_{A^{\prime }}-\frac{x_{A}+x_{O}}{2}\\ y_{A^{\prime }}-\frac{y_{A}+y_{O}}{2} \end{array}\right]$$

That is, if the radius of the trajectory is much larger than the moving distance, R≫l, point $B\left(x_{B},y_{B}\right)$ can be approximated as the midpoint of the ideal movement segment OA.

Substituting the prototype parameters and the ideal forward moving distance l=1000 mm into Equation (46), the included angle between the ideal ending direction vector $\vec{OA}$ and the actual ending direction vector $\vec{BA^{\prime }}$ is 0.0416 rad (2.381°).

## 5. Estimation of Error Distribution on Multi-Point Path Motion

According to Equation (3), as the parameters of the error model are found, shown in Table 1, the optimal route with the least error can be calculated. In this section, the theoretical precision optimal path is first proposed.

However, the error parameter is variable. A simulation with individual variable error parameters was made to determine the relationship between the parameter changing and the error changing.

Based on the variant parameters’ simulation, as the rotation error affects the average error the most, the path with the smallest summation angle has the least error. A prototype experiment validates the model.

### 5.1. Precision Optimal Path with Invariant Measured Error Parameters

Substitute the recognized error parameters shown in Table 1. The precision optimal path can be calculated by the genetic algorithm. The precision-driven optimal path is shown in Figure 14a. The theoretical position error comparison with three characteristic paths is shown in Figure 14b.

As shown in Figure 14a, the points in red represent the ideal target positions, and the points and path in blue represent the simulated motion path with recognized error parameters shown in Table 1.

Figure 14b shows the error comparisons among the three characteristic paths. The abscissa axis denotes the sequence of target points, and the ordinate axis denotes the position error at each target point.

The theoretical average error for the optimal precision path is 32.2795 mm. However, compared with the other paths, the average error of the shortest-length path is 46.3663 mm, and the average error of the least rotation path is 42.3186 mm. The precision optical path performs better than the other paths. What is more, the proposed precision optimal path has the best precision among all paths with any sequence of target points according to the calculated processing of the genetic algorithm.

### 5.2. Simulation with Variant Error Parameters

However, the error parameters are not constant at different movements. Along with the random change in the error parameters, the motion performances vary after every movement. Each parameter is considered to fit the normal distribution. The true position at every target point must fit a specific distribution. A simulation was made to show this distribution.

**Figure 15:**
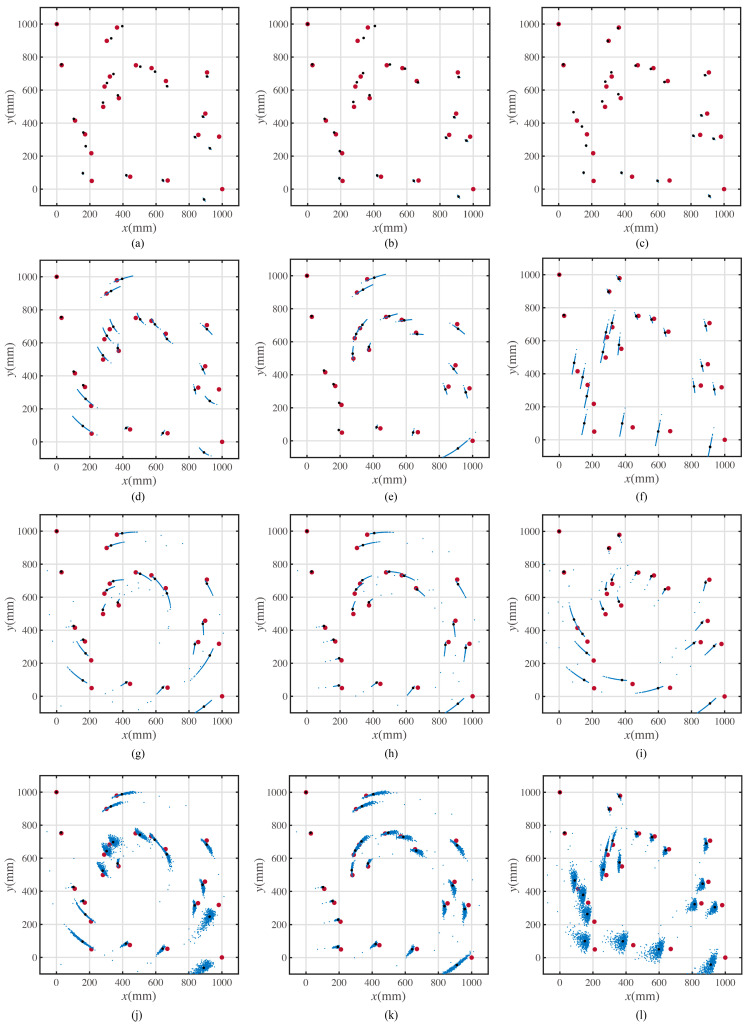
Simulated positions with different path and error parameters. (**a**) The simulated positions for mobile robot moving along the minimum rotation angle path with considering the forward moving error coefficient as a random variable. (**b**) The simulated positions for mobile robot moving along the minimum average position error path with considering the forward moving error coefficient as a random variable. (**c**) The simulated positions for mobile robot moving along the minimum forward moving distance path with considering the forward moving error coefficient as a random variable. (**d**) The simulated positions for mobile robot moving along the minimum rotation angle path with considering the rotation angle error coefficient as a random variable. (**e**) The simulated positions for mobile robot moving along the minimum average position error path with considering the rotation angle error coefficient as a random variable. (**f**) The simulated positions for mobile robot moving along the minimum forward moving distance path with considering the rotation angle error coefficient as a random variable. (**g**) The simulated positions for mobile robot moving along the minimum rotation angle path with considering the rotation center lateral offset as a random variable. (**h**) The simulated positions for mobile robot moving along the minimum average position error path with considering the rotation center lateral offset as a random variable. (**i**) The simulated positions for mobile robot moving along the minimum forward moving distance path with considering the rotation center lateral offset as a random variable. (**j**) The simulated positions for mobile robot moving along the minimum rotation angle path with considering all error parameters are random variables. (**k**) The simulated positions for mobile robot moving along the minimum average position error path with considering all error parameters are random variables. (**l**) The simulated positions for mobile robot moving along the minimum forward moving distance path with considering all error parameters are random variables.

In the numerical simulations, each error parameter can be set as a random variable or a constant. When considering any error parameter as a constant, its value follows the value shown in Table 1. When considering any error parameter as a random variable, it fits a normal distribution with the same mean value shown in Table 1, and the same standard deviation shown in Table 1. Moreover, each simulation is made with different paths. The two characteristic paths are the minimum forward moving distance path and the minimum rotation angle path. Besides these two paths, the optimal precision path is simulated to show its superiority in precision. The results of the simulations are shown in Figure 15.

In each subfigure of Figure 15, the biggest points in red represent the ideal positions. The medium-size points in black represent the theoretical position when considering the error parameters as constant. The smallest points in blue represent the possible positions when considering the error parameters as the random variables. When considering the parameters as random variables, the distributions of the variables are seen as the normal distribution. The parameters of the normal distributions fit the value shown in Table 1. The simulations were repeated five hundred times.

The possible positions scatter points of the minimum rotation angle path with only the forward moving error coefficient $k_{s}$ variable are shown in Figure 15a. The possible positions of the scatter points of the minimum average-position-error path with only the forward moving error coefficient $k_{s}$ variable are shown in Figure 15b. The possible positions of the scatter points of the minimum forward moving distance path with only the forward moving error coefficient $k_{s}$ variable are shown in Figure 15c.

The possible positions of the scatter points of the minimum rotation angle path with only the rotation angle error coefficient $k_{r}$ variable are shown in Figure 15d. The possible positions of the scatter points of the minimum average-position-error path with only the rotation angle error coefficient $k_{r}$ variable are shown in Figure 15e. The possible positions of the scatter points of the minimum forward moving distance path with only the rotation angle error coefficient $k_{r}$ variable are shown in Figure 15f.

The possible positions of the scatter points of the minimum rotation angle path with only the rotation center lateral offset $d_{r}$ variable are shown in Figure 15g. The possible positions of the scatter points of the minimum average-position-error path with only the rotation center lateral offset $d_{r}$ variable are shown in Figure 15h. The possible positions of the scatter points of the minimum forward moving distance path with only the rotation center lateral offset $d_{r}$ variable are shown in Figure 15i.

The possible positions of the scatter points of the minimum rotation angle path with all error parameters are variable, as shown in Figure 15j. The possible positions of the scatter points of the minimum average-position-error path with all error parameters are variable, as shown in Figure 15k. The possible positions of the scatter points of the minimum forward moving distance path with all error parameters are variable, as shown in Figure 15l.

According to Figure 15, when the rotation angle error coefficient $k_{r}$ and the rotation center lateral offset $d_{r}$ are considered random variables, the position distribution at each target point has greater dispersion.

### 5.3. Prototype Experiment and the Comparison with the Simulated Result

**Figure 16:**
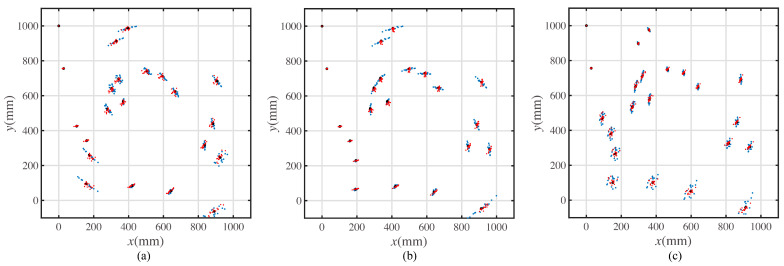
Position scatters of simulated results and experimental results. (**a**) The simulated and experimental positions for mobile robot moving along the minimum rotation angle path. (**b**) The simulated and experimental positions for mobile robot moving along the minimum average position error path. (**c**) The simulated and experimental positions for mobile robot moving along the minimum forward moving distance path.

A prototype experiment was conducted to validate the proposed error model. The prototype was the same as the mobile robot shown in Figure 8. The experimental paths are the characteristic paths shown in Figure 3b,c and Figure 14a. The motion on each path was repeated fifteen times. The result and comparison with the simulated results is shown in Figure 16.

In Figure 16, the blue points represent the simulation’s calculated points, and the points in red represent the points from the prototype experiment.

Figure 16a shows the results comparison of the motion path with the least rotation. Figure 16b shows the results comparison of the motion path with the optimal precision. Figure 16c shows the results comparison of the motion path with the shortest length.

To align the experimental coordinates captured by the motion capture system, the actual start point of the mobile robot is (0,1100), and the initial pose is (0,−1). The movement of a mobile robot moving from the point (0,1100) to the start point of the path (0,1000) is called the direction calibration movement. The transformations were built to make the coordinates of the start point coincide with the ideal start point (0,1000), and the transformation angle is determined by the direction calibration movement.

The points in black represent the simulated points with the constant error coefficients. As shown in Figure 16, the experimental points are located around the simulated points.

The data shown in Table 2 verify that the error model simulates the error of the experimental positions compared with the ideal target point positions. The position error is defined as the distance between the theoretical or experimental position and the ideal target position.

As shown in Table 2, the position error generally increases when moving along the path. The original experiment data are available in Table A5, Table A6 and Table A7.

Each error parameter is supposed to fit the normal distribution. The experiment results shown in Table 2 validate that the mean values of the distributions are rational. What is more, the experiment results validate that the proposed three-parameter model can reflect the error of the mobile robot movements.

Considering the error parameters as random variables, the position distribution of the experimental mobile robot at each ideal target position was tested with the same distribution of the simulated positions. The average position error of the entire path is shown in Table 3.

As shown in Table 3, the average position error is the smallest when moving along the path of minimum theoretical error. The path with the minimum forward moving distance corresponds to the largest position error. What is more, the average experimental position errors are smaller than the simulated errors. This may be because the error parameters are seen as completely random. However, the error parameter of adjacent movements may have relative values.

## 6. Optimal Path with Command Compensation of the Mobile Robot

If the error parameters are known, compensating the motion commands helps to decrease the position error. The movement mode in this article is considered the dual mode. There are only two types of movement, the forward movement and rotation in situ. Corresponding to these two movement types, the forward moving distances, $l_{i}$, and the rotation angles, $\theta_{i}$, comprise the entire movement commands.

The compensation for the forward moving distance is shown in Equation (48).

(48) $$l_{i}^{*}=\frac{l_{i}}{k_{s}}$$

where $l_{i}^{*}$ represents the actual forward moving distance command after compensation, $l_{i}$ represents the ideal forward moving distance when considering there is no movement error, and $k_{s}$ represents the forward moving error coefficient.

The compensation for the rotation angle is shown in Equation (49).

(49) $$\theta_{i}^{*}=\frac{\theta_{i}-\frac{2l_{i}d_{r}}{w^{2}}-\alpha_{i-1}}{k_{r}}$$

where $\theta_{i}^{*}$ represents the actual rotation angle command after compensation, $\theta_{i}$ represents the ideal rotation angle when considering there is no movement error, $l_{i}$ still represents the ideal forward moving distance when considering there is no movement error, $d_{r}$ represents the rotation center lateral offset, w represents the wheel track of the mobile robot, $k_{r}$ represents the rotation angle error coefficient, and $\alpha_{i-1}$ represents the initial pose error caused by the last forward movement.

(50) $$\alpha_{i-1}=\arccos\left(\frac{2-\cos\left(\frac{2l_{i-1}d_{r}}{w^{2}}\right)}{\sqrt{5-4\cos\left(\frac{2l_{i-1}d_{r}}{w^{2}}\right)}}\right)$$

The initial condition of Equation (50) is

(51) $$\alpha_{1}=0$$

**Figure 17:**
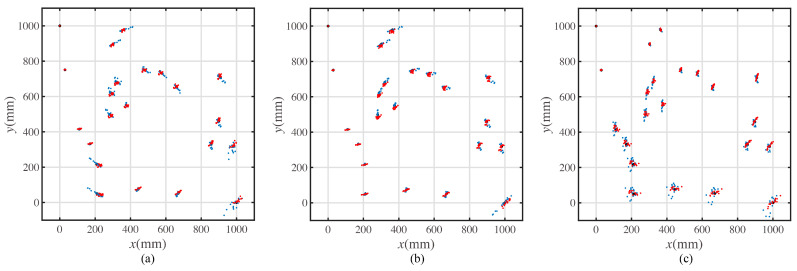
Movement precision experiment with different paths. (**a**) The simulated and experimental positions for mobile robot moving along the minimum rotation angle path with command compensation. (**b**) The simulated and experimental positions for mobile robot moving along the minimum average position error path with command compensation. (**c**) The simulated and experimental positions for mobile robot moving along the minimum forward moving distance path with command compensation.

The position errors are expected to decrease by compensating for every movement command. The result of the prototype experiment is shown in Figure 17. The simulated positions are also shown in Figure 17.

In Figure 17, the points in blue represent the simulated positions, the points in red represent the experimental positions, and the points in black represent the ideal target positions almost coincident with the experimental position points.

As shown in Figure 17, the error, in reality, is coincident with the simulated positions. The data in Table 4 show the error of the mobile robot after command compensation.

If considering the error parameters as constants, the theoretical simulated position errors are all zero. Comparing the data in Table 2 and Table 4, the compensations of the commands reduce the position error. The original experiment data are available in Table A8, Table A9 and Table A10.

The average position error data of the simulation and experiment are shown in Table 5.

Moving along the minimum error path still has the minimum average position error, and the minimum forward moving distance path corresponds to the largest error. However, the value differences between different paths decrease from the non-compensation experiment.

The prototype experiment verifies that the precision optimal path calculated by the proposed method has better precision.

## 7. Discussion

Although the structure of the mobile robot shown in Figure 2 is a four-Mecanum-wheel mobile robot, the proposed method of error estimation and precision-driven path planning can be applied to any mobile robot whose motion mode is moving forward and rotation in situ.

The controlling strategy design is going to be universal in robot integration. The integration system developer uses the mobile robot with an entire submodule. It is difficult to adjust the detail of the controlling program of each wheel. Using only the forward moving command and rotation command is common in the integration robot system.

The proposed three-parameter error model contains the forward moving error coefficient, $k_{s}$, the rotation angle error coefficient, $k_{r}$, and the rotation center lateral offset, $d_{r}$. The forward moving error coefficient, $k_{s}$, reflects one of the errors in forward movement. The actual moving distance is considered linear to the ideal forward moving distance. The distribution of this coefficient was tested as an approximately normal distribution, as shown in Figure 12.

The rotation angle error coefficient, $k_{r}$, reflects the error of the rotation movement. The actual rotation angle is considered linear to the ideal rotation angle. The distribution of this coefficient was tested as an approximately normal distribution, as shown in Figure 12.

The rotation center lateral offset, $d_{r}$, reflects the pose error caused by the forward movement. The trajectory shape of forward movement is considered arc-shaped. The distribution of $d_{r}$ is estimated by the arc-shaped trajectory fitting radius. The transformation from the fitted radius to the error parameter $d_{r}$ is shown in Equation (17).

We only need to substitute the nominal value for the wheel track w. That is because for every use of $d_{r}$, $1/w^{2}$ comprises the coefficient of $d_{r}$. No matter the value of w, the value of $d_{r}/w^{2}$ is changeless.

The random sequence path movements were used to collect data for estimating the error parameters. All the simulations and experiments used the target points shown in Table A1. The actual moving distance and rotating angle were captured by the motion capture system. However, these realistically observed variables can be calculated by other sensor systems. In contrast to the offline estimation proposed in this article, the parameters’ estimation can be made during the movement, called the online parameters’ estimation. The data of offline estimation are sufficient. As shown in Table A2, Table A3 and Table A4, hundreds of data are used to estimate the error parameters. The number of historical movement data in online estimation is probably less than that in offline estimation. The accuracy of online estimation will be lower than that of offline; therefore, offline estimation is sufficient.

The processes of $d_{r}$ estimation can be improved in future studies. In this study, the radius of the trajectory was fitted first. Then, the distribution of the rotation center lateral offset, $d_{r}$, was fitted according to the dataset of radii. The fitting of $d_{r}$ can be directly calculated from the trajectory data captured by the motion captured system. However, the value of $d_{r}$ estimated in this article already matches the experimental results, as shown in Figure 16 and Figure 17. The simulated positions, which use the fitted value $d_{r}$, are close to the experimental positions.

When considering the randomness of the error parameters, the position distribution at each target point is different between the simulated and experimental results. As shown in Figure 16 and Figure 17, the distribution of the simulated results has more randomness than that of the experimental results. This is because the error parameters of adjacent movements may have a relative value in reality, and the prototype experiments are conducted continuously. This also causes the relative value of the error parameters. Conversely, the error parameters are considered totally random in the simulation.

There are many other factors that affect the error of the movements. Firstly, the ground conditions cannot be uniform everywhere. The contact condition between the wheel and the ground affects the error parameters. Secondly, the mechanical clearances at the moment of starting and stopping generate the random motion error. Thirdly, the vibration causes unpredictable motion error. Finally, the trajectories captured by the motion capture system cannot be precise. However, these factors are less of an influence than the influence of the proposed three parameters, $k_{s}$, $k_{r}$, and $d_{r}$.

If the mobile robot moves along the path sequence with the minimum position error, the expected average error is the minimum. The separate simulation is shown in Figure 15a–c; the impact of the forward moving error coefficient, $k_{s}$, is less on the position error. The numerical fluctuations in $k_{r}$ and $d_{r}$ cause obvious differences in position error. Hence, the path with the minimum position error and the path with the minimum rotation angle are expected to have a smaller position error than that of the shortest path length.

As shown in Table 2 and Table 4, the position error does not continuously increase with the sequence of the path. This property guides the strategy for the localization system. The localization only needs to be performed at the positions with the higher error.

**Figure 18:**
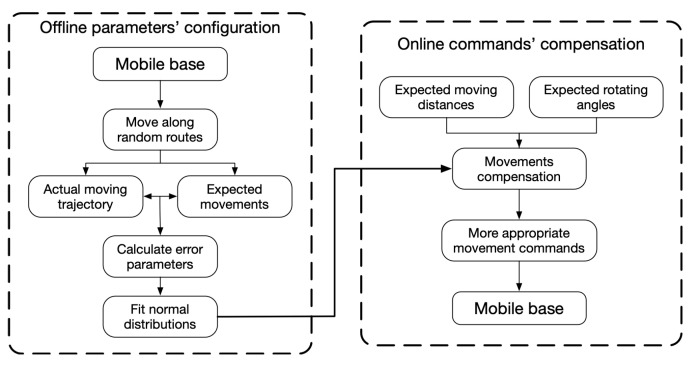
Flowchart for the movement command compensation.

Figure 18 shows the processes of utilizing the proposed error model to obtain a higher localization precision by odometry. The processes are divided into two parts, the offline parameters’ identification and the online commands’ compensation. If the error parameters of a mobile base are unknown, let the mobile base move along random routes and record the actual moving trajectory. The error parameters can be calculated according to this article’s proposed model. The expected initial movements are also required in the parameter calculation. The fitted distributions of the error parameters are used for the online motion compensation.

The movement types for multi-target path tracking are moving forward and rotating. The expected moving distances and the expected rotating angles can be compensated to obtain a higher localization precision by the equations proposed in Section 6.

## 8. Conclusions

This article first proposed a method to solve the precision-driven multiple-target path planning problem. If a precision function can estimate the localization error of the path with a certain routing sequence, the proposed method can find the optimal routing sequence with the lowest error. Localization error is defined as the distance between the actual and ideal target positions. Moreover, the precision-driven multi-target path planning method works only for mobile robots moving with approximate, known and systematic errors.

A three-parameter odometry error model based on the dual-mode movements was then proposed. The dual modes are the forward movement and the rotation in situ. This model includes three parameters, the forward moving error coefficient, $k_{s}$, the rotation angle error coefficient, $k_{r}$, and the rotation center lateral offset, $d_{r}$. Based on these parameters, the true positions affected by the error parameters can be estimated by a set of actual movement equations.

As each position error can be known according to the specific sequence of target positions, the minimum precision path with the optimal sequence is calculated based on the minimum average position error criterion.

A prototype experiment was designed to validate the proposed error model. The simulated position data matches the experimental position data well. On the prototype, the error parameters can be identified by analyzing the random movements’ actual trajectory. The distribution of the parameters fit the normal distribution well.

As the error parameters are known, the movement commands can be compensated to obtain a high-precision localization in the multi-target path tracking. The prototype experiment results validate the command compensation. The position error is reduced after the compensation. Comparing the three characteristic paths, the path with the minimum theoretical position error was tested with the least position error. What is more, the rotation angle error coefficient, $k_{r}$, and the rotation center lateral offset, $d_{r}$, take higher weight on affecting the position error. When the exact value of the error parameters is unknown, moving along the path with the least rotation angle has a lower expected position error than the path with the shortest length.

## Figures and Tables

**Table 1:** Error parameters estimated by the experiment.

Error Parameters	Mean Value	Standard Deviation
$k_{s}$	0.975887	0.0029193
$k_{r}$	1.00063	0.0081595
$d_{r}$	0.14965 mm	0.07293 mm

**Table 2:** Experimental validation of position error with proposed three-parameter error model (mm).

Path with the Least Rotation				Path with Optimal Precision				Path with the Shortest Length			
Position Sequence	Simulated Error	Average Experimental Error	Δd	Position Sequence	Simulated Error	Average Experimental Error	Δd	Position Sequence	Simulated Error	Average Experimental Error	Δd
1	0.00	0.00	0	1	0.00	0.00	0	1	0.00	0.00	0
2	5.11	6.50	−1.39	2	5.11	5.61	−0.5	2	5.11	6.40	−1.29
3	13.04	15.44	−2.4	3	13.04	12.84	0.2	3	5.47	5.28	0.19
4	15.27	17.36	−2.09	4	15.27	14.28	0.99	4	6.51	6.31	0.2
5	24.87	29.63	−4.76	5	18.80	17.43	1.37	5	13.34	15.14	−1.8
6	28.89	33.97	−5.08	6	24.71	24.81	−0.1	6	16.31	16.90	−0.59
7	24.35	29.18	−4.83	7	24.07	20.98	3.09	7	20.93	22.31	−1.38
8	23.43	28.01	−4.58	8	28.71	26.15	2.56	8	28.64	29.75	−1.11
9	24.56	26.45	−1.89	9	25.25	23.14	2.11	9	36.70	39.27	−2.57
10	32.95	25.20	7.75	10	32.61	32.23	0.38	10	46.17	49.53	−3.36
11	30.76	21.10	9.66	11	24.86	22.69	2.17	11	41.35	43.77	−2.42
12	18.12	14.13	3.99	12	28.67	30.82	−2.15	12	27.88	33.75	−5.87
13	71.13	55.97	15.16	13	10.81	17.29	−6.48	13	25.42	33.21	−7.79
14	54.49	40.28	14.21	14	15.60	21.90	−6.3	14	30.21	36.54	−6.33
15	25.95	16.84	9.11	15	19.55	23.55	−4	15	37.29	43.12	−5.83
16	25.79	17.80	7.99	16	43.59	44.53	−0.94	16	54.98	63.50	−8.52
17	26.47	19.87	6.6	17	40.29	37.92	2.37	17	55.99	62.16	−6.17
18	27.65	26.84	0.81	18	25.27	21.66	3.61	18	62.90	70.22	−7.32
19	29.91	31.02	−1.11	19	27.23	21.20	6.03	19	78.52	88.09	−9.57
20	32.29	36.18	−3.89	20	30.14	20.56	9.58	20	67.95	72.71	−4.76
21	89.88	90.25	−0.37	21	18.07	11.98	6.09	21	72.45	78.00	−5.55
22	126.41	124.06	2.35	22	98.74	82.36	16.38	22	97.97	109.08	−11.11

**Table 3:** Average position error of simulation and experiment (mm).

Type of Path	Average Error of Simulated Positions	Average Error of Experimental Positions
Minimum rotation angle path	37.12 mm	32.09 mm
Minimum theoretical error path	27.42 mm	24.27 mm
Minimum forward moving distance path	43.82 mm	42.05 mm

**Table 4:** Average position error at each target position (mm).

Position Sequence	Path with the Least Rotation	Path with Optimal Precision	Path with the Shortest Length
1	0.00	0.00	0.00
2	2.40	2.36	2.25
3	6.23	5.91	3.89
4	7.35	6.75	4.59
5	10.60	7.51	7.22
6	12.55	8.41	8.48
7	12.44	8.74	9.64
8	12.18	10.12	11.95
9	12.52	9.90	13.98
10	8.74	11.25	16.19
11	9.27	10.15	15.01
12	8.91	10.05	8.83
13	21.34	8.59	8.47
14	19.54	8.86	7.98
15	13.45	8.92	9.66
16	11.74	12.39	13.49
17	10.74	13.45	14.17
18	8.30	14.28	16.10
19	8.07	14.90	19.26
20	8.85	16.12	18.28
21	13.70	16.09	19.41
22	17.51	22.19	23.43

**Table 5:** Average error of simulation and experiment (mm).

Type of Path	Average Error of Simulated Positions	Average Error of Experimental Positions
Minimum rotation angle path	13.36 mm	10.75 mm
Minimum theoretical error path	11.34 mm	10.32 mm
Minimum forward moving distance path	15.64 mm	11.47 mm

## Data Availability

Not applicable.

## Appendix A

The coordinates of the target points are shown in Table A1. Every simulation and experiment proposed in this article used the points shown in Table A1.

**Table A1:** Coordinates of the target points (mm).

Number	*x*	*y*	Number	*x*	*y*	Number	*x*	*y*
1	280.55	498.39	9	208.69	217.62	17	572.88	733.00
2	478.30	750.55	10	908.04	707.02	18	659.59	654.93
3	364.05	979.02	11	443.71	75.28	19	29.41	751.03
4	302.14	898.32	12	110.49	415.21	20	375.17	551.08
5	211.25	49.67	13	320.87	682.16	Start	0	1000
6	171.07	332.16	14	981.18	317.89	End	1000	0
7	670.33	52.25	15	288.61	621.57			
8	855.58	328.63	16	897.85	457.72			

## Appendix B

Four random paths with random sequences of the target points were generated for estimating the error parameters of the proposed error model. The paths are shown in Figure A1.

In Figure A1, the points in black represent the target positions. The lines in red represent the moving path for mobile robot. The correspondence data between the expected movement distances and actual movement distances are shown in Table A2. The correspondence data between the expected rotation angles and actual rotation angles are shown in Table A3. The fitted radius data of the forward-moving trajectory are shown in Table A4. Only the trajectories whose captured pulse lengths were larger than 1500 were used for fitting the trajectory radius.

**Table A2:** Expected movement distances and actual movement distances (mm).

Ideal Distance	True Distance	Ideal Distance	True Distance	Ideal Distance	True Distance	Ideal Distance	True Distance	Ideal Distance	True Distance	Ideal Distance	True Distance	Ideal Distance	True Distance	Ideal Distance	True Distance	Ideal Distance	True Distance
100	97.79	809.73	789.08	631.07	615.79	631.07	615.78	602.78	585.86	100	96.73	973.53	952.52	100	98.21	1048.9	1022.81
809.73	790.71	631.07	616.99	670.29	654.97	670.29	654.12	592.43	578.50	100	98.04	285.34	278.90	973.53	948.56	729.79	709.58
631.07	617.23	670.29	655.86	476.02	462.47	476.02	463.60	723.51	708.75	595.13	581.54	810.24	787.61	285.34	278.12	656.35	640.54
670.29	654.06	476.02	463.22	667.21	651.28	667.21	648.87	318.45	313.54	476.02	465.10	589.69	573.47	810.24	789.58	641.02	624.25
476.02	462.84	667.21	648.83	696.59	679.78	696.59	679.45	595.13	581.33	676.15	662.62	567.89	553.35	589.69	575.55	339.82	329.97
667.21	650.07	696.59	679.30	625.29	608.53	625.29	607.08	476.02	464.07	96.193	93.28	411.78	402.62	567.89	553.03	438.61	427.65
696.59	679.20	625.29	609.26	289.73	281.09	289.73	282.69	676.15	658.84	543.77	530.22	490.37	478.13	411.78	400.16	428.08	417.77
625.29	608.04	289.73	282.28	724.55	707.60	724.55	707.27	543.77	531.20	927.89	905.36	720.36	703.90	490.37	477.49	268.68	262.32
289.73	282.24	724.55	706.41	753.23	733.93	753.23	734.65	927.89	907.62	638.18	622.81	339.82	330.63	720.36	701.77	567.57	553.56
724.55	706.59	753.23	736.95	585.52	568.96	585.52	571.51	638.18	624.41	277.07	270.03	317.22	309.09	339.82	331.84	312.37	305.06
753.23	736.06	585.52	571.70	519.08	506.15	519.08	507.22	277.07	271.23	141.88	139.54	354.83	347.51	116.68	114.03	625.29	608.59
585.52	573.51	519.08	506.99	663.38	648.65	663.38	647.37	101.71	100.01	530.96	520.61	530.96	517.91	317.22	308.99	661.26	644.83
519.08	507.26	663.38	647.41	126.05	122.46	126.05	122.80	299.98	293.03	798.71	781.08	618.64	602.83	354.83	348.12	592.43	576.84
663.38	647.33	126.05	122.84	794.26	773.71	794.26	776.27	530.96	519.52	957.93	934.81	661.26	644.00	530.96	516.47	408.89	397.36
794.26	775.83	794.26	775.10	101.71	100.13	101.71	99.93	798.71	780.83	853.58	834.82	879.73	857.44	618.64	601.94	815.31	793.78
428.08	419.41	428.08	418.84	428.08	418.46	428.08	418.78	957.93	933.01	120.56	117.08	927.89	904.45	661.26	644.92	853.51	832.41
141.88	139.70	618.64	602.72	141.88	138.76	188.14	185.11	853.58	832.37	585.52	570.97	566	551.51	879.73	858.11	835.13	813.66
188.14	185.03	468.97	458.36	188.14	183.44	618.64	602.12	120.56	117.86	602.78	589.04	497.73	484.64	927.89	906.33	792.64	772.84
618.64	603.59	100	98.17	618.64	603.32	468.97	458.14	585.52	572.27	592.43	576.30	618.2	603.26	566	552.30	448.89	439.12
468.97	456.55	100	96.90	468.97	458.08	100	97.29	602.78	588.03	723.51	707.40	1167.4	1139.15	497.73	486.37	497.73	485.22
100	97.41	809.73	790.55	100	97.76	100	98.21	592.43	576.32	100	97.50	100	97.63	618.2	604.82	981.65	955.33
100	98.20	631.07	616.47	100	98.24	595.13	582.76	723.51	706.37	595.13	580.83	973.53	949.59	1167.4	1138.42	1048.9	1023.95
809.73	791.37	670.29	657.43	809.73	788.88	476.02	465.07	318.45	313.15	476.02	465.33	285.34	277.28	973.53	949.63	729.79	710.29
631.07	615.97	476.02	462.89	631.07	616.27	676.15	661.24	595.13	580.55	676.15	657.86	810.24	789.93	285.34	277.27	656.35	639.56
670.29	653.41	667.21	649.63	670.29	656.92	543.77	532.61	476.02	465.09	543.77	531.23	589.69	573.31	810.24	791.17	641.02	626.48
476.02	463.56	696.59	680.01	476.02	462.61	927.89	905.21	676.15	660.60	927.89	906.01	567.89	554.50	589.69	575.02	339.82	330.34
667.21	651.67	625.29	609.64	667.21	650.38	638.18	623.11	96.193	93.47	638.18	625.20	411.78	401.68	567.89	552.81	438.61	429.20
696.59	679.86	289.73	283.36	696.59	682.01	277.07	272.40	543.77	529.48	277.07	271.80	490.37	477.79	411.78	400.38	428.08	417.00
625.29	609.51	724.55	706.99	625.29	608.89	299.98	291.37	927.89	906.13	101.71	99.56	720.36	702.37	490.37	476.72	268.68	262.65
289.73	283.40	753.23	737.80	289.73	282.04	530.96	519.93	638.18	625.14	299.98	292.02	339.82	329.97	720.36	702.35	567.57	554.58
724.55	707.81	585.52	573.97	724.55	706.00	798.71	779.90	277.07	270.88	141.88	139.11	116.68	114.10	339.82	331.11	312.37	303.23
753.23	734.88	519.08	507.48	753.23	734.76	957.93	936.57	101.71	99.60	530.96	518.46	317.22	308.12	354.83	347.38	625.29	609.72
585.52	571.73	663.38	648.47	585.52	569.49	853.58	832.92	299.98	292.07	798.71	777.72	354.83	346.28	530.96	515.28	661.26	645.18
519.08	505.44	126.05	122.24	519.08	506.06	120.56	117.58	141.88	138.04	957.93	934.38	530.96	516.76	618.64	600.89	592.43	576.91
663.38	648.17	794.26	775.45	663.38	647.87	585.52	570.21	530.96	520.13	853.58	831.69	618.64	601.34	661.26	643.53	408.89	398.97
126.05	123.23	101.71	99.81	126.05	123.59	602.78	589.45	798.71	779.55	120.56	117.39	661.26	645.18	879.73	856.41	815.31	791.94
794.26	774.59	428.08	419.12	794.26	772.12	592.43	578.60	957.93	932.98	585.52	571.88	879.73	858.38	927.89	904.51	853.51	832.91
101.71	100.12	618.64	601.68	101.71	99.19	723.51	705.84	853.58	834.70	602.78	589.58	927.89	904.20	566	550.48	835.13	815.25
428.08	419.01	468.97	458.74	428.08	416.09	141.88	139.48	120.56	116.95	592.43	576.43	566	551.90	497.73	486.63	792.64	771.19
188.14	183.65	100	98.44	141.88	138.82	530.96	519.40	585.52	571.15	723.51	707.51	497.73	486.35	618.2	604.23	448.89	439.08
618.64	603.18	100	97.31	188.14	184.55	798.71	779.88	602.78	587.32	100	98.14	618.2	603.06	1167.4	1138.10	497.73	486.80
468.97	456.92	809.73	791.09	618.64	603.62	957.93	934.01	592.43	576.73	973.53	952.28	1167.4	1139.92	973.53	947.39	981.65	954.97
809.73	791.82	631.07	616.34	468.97	459.06	853.58	834.89	723.51	706.67	285.34	277.37	100	97.11	285.34	276.21	1048.9	1021.95
631.07	616.63	670.29	655.47	809.73	789.05	120.56	117.48	318.45	313.15	810.24	789.63	973.53	951.80	810.24	790.20	729.79	710.41
670.29	654.97	476.02	462.45	631.07	615.92	585.52	570.52	100	97.85	589.69	575.69	285.34	279.07	589.69	573.28	656.35	638.43
476.02	462.95	667.21	649.42	670.29	656.88	602.78	589.31	595.13	582.18	567.89	554.32	810.24	788.12	567.89	552.72	641.02	626.01
667.21	650.00	696.59	679.64	476.02	463.32	592.43	575.76	476.02	465.87	411.78	401.59	589.69	573.94	411.78	402.33	339.82	332.02
696.59	680.37	625.29	610.75	667.21	649.28	723.51	706.56	676.15	658.95	490.37	479.22	567.89	553.01	490.37	476.50	438.61	426.73
625.29	610.80	289.73	284.21	696.59	682.36	595.13	581.91	543.77	529.64	720.36	704.65	411.78	401.19	720.36	700.77	428.08	418.40
289.73	281.84	724.55	708.30	625.29	608.89	476.02	464.85	927.89	906.92	339.82	330.60	490.37	478.93	339.82	331.69	268.68	263.46
724.55	707.93	753.23	733.78	289.73	282.18	676.15	659.50	638.18	624.51	116.68	114.66	720.36	703.65	116.68	114.88	567.57	554.87
753.23	734.30	585.52	570.62	724.55	705.96	96.193	94.20	277.07	271.81	317.22	310.12	339.82	330.38	317.22	307.05	312.37	302.64
585.52	569.97	519.08	506.35	753.23	735.11	543.77	529.68	299.98	292.95	354.83	347.10	317.22	308.21	354.83	346.36	625.29	608.88
519.08	505.96	663.38	649.30	585.52	571.91	927.89	906.14	141.88	138.75	530.96	519.24	354.83	347.69	530.96	515.31	661.26	646.31
663.38	649.33	126.05	122.80	519.08	504.54	638.18	625.08	530.96	519.24	618.64	600.58	530.96	517.28	618.64	601.57	592.43	576.44
126.05	123.17	794.26	775.08	663.38	647.65	277.07	269.85	798.71	780.18	661.26	645.24	618.64	605.14	661.26	643.72	408.89	398.89
794.26	774.02	101.71	100.13	126.05	123.18	299.98	292.43	957.93	934.04	879.73	857.89	661.26	645.15	879.73	857.34	815.31	795.86
101.71	98.95	428.08	418.13	794.26	774.21	141.88	139.16	853.58	833.01	927.89	905.61	879.73	857.13	927.89	905.01	853.51	833.18
428.08	417.37	141.88	139.32	101.71	98.39	530.96	517.70	120.56	117.24	566	552.36	927.89	904.48	566	551.72	835.13	815.27
141.88	139.17	618.64	603.56	428.08	417.75	798.71	780.75	585.52	573.76	497.73	486.91	566	551.96	497.73	485.67	792.64	771.82
188.14	184.96	468.97	458.27	618.64	602.39	957.93	933.28	602.78	587.67	618.2	603.56	497.73	483.31	618.2	603.32	448.89	436.06
618.64	603.01	100	97.84	468.97	460.27	853.58	831.77	592.43	578.34	1167.4	1138.59	618.2	604.72	1167.4	1138.43	497.73	485.76
468.97	457.49	100	98.27	100	98.31	120.56	118.54	723.51	707.95	100	97.39	1167.4	1139.57	100	97.23	981.65	955.18
100	97.49	809.73	790.08	809.73	788.31	585.52	572.78	318.45	311.64	100	96.92	100	96.92	100	97.71	100	98.03

**Table A3:** Expected rotation angles and actual rotation angles (rad).

Ideal Distance	True Distance	Ideal Distance	True Distance	Ideal Distance	True Distance	Ideal Distance	True Distance	Ideal Distance	True Distance	Ideal Distance	True Distance	Ideal Distance	True Distance	Ideal Distance	True Distance	Ideal Distance	True Distance
−0.261	−0.257	1.285	1.280	−1.721	−1.731	1.285	1.270	2.922	2.912	1.304	1.310	2.733	2.703	−0.995	−0.991	2.263	2.248
−2.266	−2.280	−1.351	−1.334	1.285	1.271	−1.351	−1.348	−2.996	−3.011	2.137	2.138	2.143	2.151	−3.064	−3.056	−2.976	−2.970
2.720	2.716	−0.261	−0.255	−1.351	−1.337	−0.261	−0.263	1.864	1.833	2.441	2.460	−0.995	−0.985	1.730	1.723	−2.387	−2.373
2.172	2.179	−2.266	−2.296	−2.266	−2.304	−2.266	−2.263	1.304	1.326	2.541	2.554	−0.219	−0.223	2.700	2.703	2.638	2.647
2.921	2.918	2.720	2.732	2.720	2.716	2.720	2.703	2.137	2.148	1.260	1.254	−3.064	−3.046	1.718	1.718	−2.390	−2.369
−1.798	−1.806	2.172	2.162	2.172	2.156	2.172	2.178	2.441	2.438	−1.512	−1.527	1.730	1.719	−2.670	−2.674	−2.428	−2.436
−2.056	−2.056	2.921	2.907	2.921	2.923	2.921	2.943	2.541	2.557	−2.315	−2.324	2.700	2.716	−1.422	−1.416	−2.289	−2.296
0.600	0.604	−1.798	−1.794	−1.798	−1.795	−1.798	−1.809	1.260	1.260	1.703	1.709	1.718	1.730	−2.421	−2.413	3.101	3.096
−2.288	−2.286	−2.056	−2.058	−2.056	−2.056	−2.056	−2.056	−1.512	−1.550	2.991	2.992	−2.670	−2.695	2.158	2.154	2.741	2.719
−2.250	−2.298	0.600	0.601	0.600	0.613	−2.288	−2.258	−0.589	−0.589	−2.702	−2.734	−1.422	−1.421	−2.489	−2.486	1.048	1.033
−2.792	−2.790	−2.288	−2.268	−2.288	−2.277	−2.250	−2.264	−2.315	−2.334	1.143	1.161	−2.421	−2.433	−2.326	−2.317	2.958	2.961
2.788	2.754	−2.250	−2.286	−2.250	−2.306	−2.792	−2.797	1.703	1.701	0.606	0.613	2.158	2.177	−1.187	−1.169	−1.968	−1.998
1.648	1.662	−2.792	−2.791	−2.792	−2.800	2.788	2.768	2.991	2.980	2.632	2.631	−2.489	−2.492	3.031	3.026	−1.560	−1.553
2.516	2.514	2.788	2.779	2.788	2.755	1.648	1.658	−2.702	−2.664	−0.537	−0.552	−2.326	−2.343	2.755	2.789	−2.770	−2.774
0.715	0.720	1.648	1.654	1.648	1.648	2.516	2.514	1.143	1.141	−1.001	−0.995	−1.187	−1.183	−2.771	−2.770	2.013	1.997
1.425	1.425	2.516	2.521	2.516	2.513	0.715	0.714	0.606	0.600	2.429	2.444	3.031	3.015	−2.719	−2.718	2.865	2.885
2.461	2.461	0.715	0.717	0.715	0.705	1.425	1.424	2.632	2.615	2.922	2.919	2.755	2.757	−2.773	−2.762	2.762	2.772
2.775	2.784	1.425	1.438	1.425	1.432	2.461	2.479	−0.537	−0.545	−2.996	−2.997	−2.771	−2.761	−1.580	−1.586	2.122	2.095
−2.533	−2.540	2.461	2.461	2.461	2.452	2.775	2.787	−1.001	−0.978	1.864	1.879	−2.719	−2.740	2.733	2.690	2.401	2.396
−1.721	−1.700	2.775	2.797	2.775	2.772	−2.533	−2.547	2.429	2.411	1.304	1.281	−2.773	−2.768	2.143	2.165	−1.966	−1.963
1.285	1.265	−2.533	−2.542	−2.533	−2.536	−1.721	−1.711	2.922	2.917	2.137	2.137	−1.580	−1.602	−0.995	−0.974	−0.437	−0.436
−1.351	−1.338	−1.721	−1.694	−1.721	−1.703	1.285	1.279	−2.996	−2.999	2.441	2.436	2.733	2.727	−3.064	−3.067	2.263	2.262
−0.261	−0.257	1.285	1.277	1.285	1.291	−1.351	−1.350	1.864	1.843	2.541	2.551	2.143	2.168	1.730	1.722	−2.976	−2.984
−2.266	−2.293	−1.351	−1.346	−1.351	−1.348	−0.589	−0.587	1.304	1.295	1.260	1.262	−0.995	−0.977	2.700	2.709	−2.387	−2.393
2.720	2.720	−0.261	−0.258	−0.261	−0.264	−2.315	−2.330	2.137	2.149	−1.512	−1.538	−0.219	−0.217	1.718	1.739	2.638	2.645
2.172	2.162	−2.266	−2.251	−2.266	−2.285	1.703	1.705	2.441	2.444	−0.589	−0.577	−3.064	−3.075	−2.670	−2.685	−2.390	−2.390
2.921	2.935	2.720	2.709	2.720	2.708	2.991	2.993	2.541	2.555	−2.315	−2.328	1.730	1.701	−1.422	−1.410	−2.428	−2.413
−1.798	−1.805	2.172	2.184	2.172	2.171	−2.702	−2.718	1.260	1.254	1.703	1.708	2.700	2.715	−2.421	−2.432	−2.289	−2.292
−2.056	−2.062	2.921	2.958	2.921	2.934	1.143	1.146	−1.512	−1.543	2.991	2.995	1.718	1.733	2.158	2.162	3.101	3.106
0.600	0.611	−1.798	−1.802	−1.798	−1.798	0.606	0.608	−0.187	−0.191	−2.702	−2.725	−2.670	−2.689	−2.489	−2.482	2.741	2.734
−2.288	−2.259	−2.056	−2.064	−2.056	−2.069	2.632	2.654	−0.589	−0.596	1.143	1.148	−1.422	−1.416	−0.185	−0.185	1.048	1.064
−2.250	−2.303	0.600	0.601	0.600	0.593	−1.001	−1.001	−2.315	−2.317	0.606	0.611	−2.421	−2.418	−2.326	−2.315	2.958	2.969
−2.792	−2.796	−2.288	−2.263	−2.288	−2.272	2.429	2.429	1.703	1.722	2.632	2.651	2.158	2.174	−1.187	−1.170	−1.968	−1.963
2.788	2.778	−2.250	−2.279	−2.250	−2.264	2.922	2.909	2.991	2.991	−0.537	−0.543	−2.489	−2.499	3.031	3.035	−1.560	−1.556
1.648	1.653	−2.792	−2.799	−2.792	−2.804	−2.996	−3.000	−2.702	−2.713	−1.001	−0.992	−2.326	−2.317	2.755	2.763	−2.770	−2.786
2.516	2.519	2.788	2.796	2.788	2.758	1.864	1.842	1.143	1.146	2.429	2.421	−1.187	−1.183	−2.771	−2.771	2.013	2.017
0.715	0.716	1.648	1.672	1.648	1.663	1.304	1.295	0.606	0.613	2.922	2.925	3.031	3.044	−2.719	−2.721	2.865	2.852
1.425	1.403	2.516	2.507	2.516	2.517	2.137	2.142	2.632	2.628	−2.996	−2.999	2.755	2.748	−2.773	−2.780	2.762	2.747
2.461	2.462	0.715	0.723	1.425	1.414	2.441	2.454	−0.537	−0.526	1.864	1.866	−2.771	−2.764	−1.580	−1.582	2.122	2.090
2.775	2.772	1.425	1.427	2.461	2.473	2.541	2.565	−1.001	−1.007	1.304	1.314	−2.719	−2.714	2.733	2.723	2.401	2.384
−2.533	−2.519	2.461	2.453	2.775	2.788	1.260	1.254	2.429	2.424	2.137	2.135	−2.773	−2.777	2.143	2.155	−1.966	−1.982
−1.721	−1.698	2.775	2.757	−2.533	−2.533	−1.512	−1.522	2.922	2.935	2.441	2.447	−1.580	−1.583	−0.995	−0.991	−1.027	−1.055
1.285	1.270	−2.533	−2.536	−1.721	−1.724	−1.001	−0.986	−2.996	−2.999	2.541	2.539	2.733	2.713	−3.064	−3.095	2.263	2.257
−1.351	−1.339	−1.721	−1.710	1.285	1.262	2.429	2.414	1.864	1.843	1.260	1.251	2.143	2.142	1.730	1.711	−2.976	−2.977
−0.261	−0.264	1.285	1.272	−1.351	−1.340	2.922	2.921	1.304	1.309	−1.512	−1.543	−0.995	−0.983	2.700	2.699	−2.387	−2.387
−2.266	−2.286	−1.351	−1.335	−2.266	−2.294	−2.996	−2.987	2.137	2.147	−0.219	−0.219	−3.064	−3.070	1.718	1.726	2.638	2.646
2.720	2.719	−2.266	−2.304	2.720	2.721	1.864	1.853	2.441	2.457	−3.064	−3.073	1.730	1.719	−2.670	−2.666	−2.390	−2.377
2.172	2.173	2.720	2.712	2.172	2.173	1.304	1.298	2.541	2.541	1.730	1.712	2.700	2.703	−1.422	−1.433	−2.428	−2.412
2.921	2.924	2.172	2.173	2.921	2.920	2.137	2.135	1.260	1.257	2.700	2.719	1.718	1.713	−2.421	−2.438	−2.289	−2.291
−1.798	−1.797	2.921	2.949	−1.798	−1.824	2.441	2.450	−1.512	−1.545	1.718	1.729	−2.670	−2.669	2.158	2.155	3.101	3.101
−2.056	−2.064	−1.798	−1.801	−2.056	−2.065	2.541	2.559	−0.589	−0.587	−2.670	−2.681	−1.422	−1.417	−2.489	−2.487	2.741	2.737
0.600	0.617	−2.056	−2.064	0.600	0.602	1.260	1.253	−2.315	−2.339	−1.422	−1.422	−2.421	−2.419	−0.185	−0.188	1.048	1.043
−2.288	−2.281	0.600	0.611	−2.288	−2.261	−1.512	−1.506	1.703	1.701	−2.421	−2.421	2.158	2.158	−2.326	−2.317	2.958	2.981
−2.250	−2.262	−2.288	−2.265	−2.250	−2.278	−0.589	−0.573	2.991	3.009	2.158	2.163	−2.489	−2.503	−1.187	−1.174	−1.968	−1.990
−2.792	−2.789	−2.250	−2.282	−2.792	−2.795	−2.315	−2.321	−2.702	−2.720	−2.489	−2.492	−2.326	−2.330	3.031	3.012	−1.560	−1.554
2.788	2.757	−2.792	−2.807	2.788	2.770	1.703	1.711	1.143	1.135	−0.185	−0.184	−1.187	−1.162	2.755	2.763	−2.770	−2.772
1.648	1.662	2.788	2.788	1.648	1.672	2.991	2.989	0.606	0.605	−2.326	−2.315	3.031	3.023	−2.771	−2.776	2.013	2.027
2.516	2.516	1.648	1.665	2.516	2.506	−2.702	−2.704	2.632	2.612	−1.187	−1.191	2.755	2.772	−2.719	−2.718	2.865	2.866
0.715	0.715	2.516	2.513	0.715	0.730	1.143	1.152	−0.537	−0.543	3.031	3.026	−2.771	−2.769	−2.773	−2.774	2.762	2.753
1.425	1.440	0.715	0.721	1.425	1.427	0.606	0.612	−1.001	−0.993	2.755	2.760	−2.719	−2.740	−1.580	−1.577	2.122	2.121
2.461	2.441	1.425	1.434	2.461	2.461	2.632	2.633	2.429	2.431	−2.771	−2.763	−2.773	−2.777	2.733	2.713	2.401	2.397
2.775	2.770	2.461	2.455	2.775	2.772	−0.537	−0.543	2.922	2.912	−2.719	−2.715	−1.580	−1.576	2.143	2.159	−1.966	−1.990
−2.533	−2.542	2.775	2.755	−2.533	−2.534	−1.001	−0.991	−2.996	−3.003	−2.773	−2.778	2.733	2.719	−0.995	−1.010		
−1.721	−1.679	−2.533	−2.537	−1.721	−1.697	2.429	2.427	1.864	1.874	−1.580	−1.593	2.143	2.180	−1.027	−1.053		

**Table A4:** Fitted radius of forward-moving trajectory (mm).

Fitted Radius	Fitted Radius	Fitted Radius	Fitted Radius	Fitted Radius	Fitted Radius	Fitted Radius	Fitted Radius	Fitted Radius	Fitted Radius
−28,029.7	−19,449.0	−33,788.8	−11,430.1	−25,462.9	−25,215.0	−8146.3	−13,963.1	−14,818.2	−8376.2
−22,911.8	−31,819.5	−22,466.3	−22,694.8	−11,062.5	−35,623.9	−31,148.0	−3123.0	−30,178.9	−8405.2
−24,242.3	−26,716.3	−4471.8	−23,169.3	−12,850.5	−34,830.9	−21,131.2	−20,201.8	−18,567.2	−13,766.4
−18,521.0	−22,575.7	−32,676.7	−29,530.8	−21,853.9	−23,692.1	−33,997.5	−27,544.4	−23,634.2	−13,299.6
−30,536.7	−17,972.2	−24,614.5	−17,959.6	−24,801.8	−19,994.6	−21,647.1	−36,128.8	−27,933.3	−38,306.7
−32,581.7	−29,239.4	−11,687.0	−29,363.3	−49,067.7	−22,690.2	−47,089.9	−37,778.0	−42,981.0	−28,086.0
−35,707.8	−30,478.8	−26,447.4	−24,564.1	−24,467.1	−25,509.8	−33,334.1	−26,938.1	−44,137.3	−28,784.3
−23,202.8	−31,694.9	−15,460.4	−31,520.8	−23,215.6	−22,998.1	−8102.5	−22,826.6	−18,737.1	−29,612.3
−33,356.9	−28,291.7	−28,084.5	−24,515.2	−26,425.0	−10,144.4	−7689.9	−6601.9	−8725.7	−25,736.0
−28,793.8	−27,341.4	−27,439.0	−16,135.8	−36,186.9	−32,230.1	−26,459.3	−12,262.2	−21,630.3	−9853.3
−2074.4	−25,432.9	−16,804.3	−23,460.3	−24,860.5	−16,176.3	−28,263.9	−24,127.8	−31,551.3	−20,570.0
−27,622.5	−15,773.2	−27,450.4	−27,708.8	−18,957.5	−43,944.0	−46,160.7	−17,913.9	−34,429.0	−25,319.2
−24,741.5	−26,772.1	−30,891.7	−13,994.0	−23,233.1	−20,809.4	−34,134.4	−23,721.6	−31,076.0	−19,690.6
−14,404.2	−24,954.1	−26,038.9	−26,855.5	−21,974.7	−12,414.0	−20,747.2	−25,837.1	−19,283.4	−17,337.4
−27,920.1	−18,643.8	−22,750.9	−12,728.9	−22,999.7	−31,198.6	−19,857.1	−45,662.1	−25,158.2	−4653.2
−16,375.3	−30,051.7	−34,217.5	−29,095.7	−22,666.4	−41,867.0	−9572.0	−42,742.6	−1709.6	−35,377.4
−25,025.4	−18,572.8	−20,947.7	−22,759.5	−23,114.4	−27,407.7	−15,143.9	−19,513.1	−16,843.3	−40,213.0
−36,564.9	−29,062.4	314.5	−26,880.4	−24,236.3	−22,158.5	−23,791.5	−23,455.2	−30,212.7	−36,098.3
−21,071.4	−25,047.0	−26,762.9	−16,121.7	−27,389.2	−19,344.9	−3486.4	−33,951.0	−13,644.6	−32,064.7
−14,536.9	−23,574.9	−25,454.7	−28,064.5	−14,956.5	−23,536.6	−24,270.6	−31,759.8	−23,367.0	−10,088.8
−30,062.3	−10,971.8	−16,692.6	−33,144.4	−14,519.5	−21,213.1	−19,052.6	−34,518.9	−19,512.1	−14,106.6
−26,450.8	−35,453.0	−27,854.4	−29,675.7	−36,844.8	−26,071.8	−42,862.3	−19,559.0	−47,778.2	−35,928.8
−16,920.9	−38,220.7	−18,893.6	−23,825.1	−20,598.8	−8665.3	−42,336.3	−17,085.8	−37,537.2	−29,202.3
−27,995.0	−26,497.3	−28,452.5	−30,894.3	−46,785.6	−39,054.1	−18,257.4	−6070.2	−19,752.7	−27,516.7
−18,383.1	−18,263.5	−8509.4	−27,409.5	−26,751.5	−19,609.9	−10,016.0	−4576.6	−14,120.9	−29,545.0
−38,311.9	−32,583.4	−22,902.0	−29,128.6	−17,356.3	−50,699.8	−22,028.4	−32,414.2	−31,735.2	−19,276.2
−27,702.4	−26,703.2	−18,375.0	−24,356.2	−32,357.1	−24,463.5	−31,424.5	−21,092.7	−28,462.7	−2848.4
−24,855.8	−30,109.1	−26,561.8	−28,256.7	−31,011.8	−19,693.7	−356.8	−27,494.3	−36,185.7	−9919.8
−17,696.5	−26,289.6	−31,696.7	−8446.7	−25,313.1	−30,729.5	−30,267.8	−21,843.3	−21,908.3	−18,675.1
−26,382.7	−11,467.0	−27,740.0	−29,160.3	−19,150.5	−37,821.0	−27,179.6	−46,309.4	−22,070.9	−20,116.4
−42,357.1	−38,478.7	−27,482.4	−20,790.4	−22,748.7	−30,800.2	−20,232.1	−39,166.7	−21,679.1	−21,634.2
−24,228.1	−17,539.3	−33,350.1	−47,320.3	−27,614.2	−22,166.1	−18,538.1	−21,155.0	−11,565.6	−20,379.7
−26,010.0	−29,688.0	−34,966.3	−21,440.9	−26,858.8	−21,441.7	−8664.0	−5385.7	−10,985.4	−5512.9
−24,303.9	−12,442.1	−7097.3	−1519.9	−29,038.4	−28,027.4	−12,142.8	−25,899.2	−14,446.1	−40,010.2
−32,753.0	−26,515.0	−30,216.8	−31,183.9	−18,401.4	−21,952.6	−28,451.5	−27,826.6	−7335.8	−31,790.8
−3302.1	−15,383.8	−29,327.6	−51,260.9	−29,124.7	−36,096.9	−18,543.8	−40,983.4	−8306.4	−30,695.9
−31,098.2	−31,479.8	−16,120.1	−25,362.1	−16,983.6	−26,573.0	−26,454.3	−37,930.5	−7459.9	−28,732.3
−28,045.8	−25,370.3	−24,241.6	−569.6	−42,419.8	−21,805.8	−23,504.9	−26,246.1	−7931.5	−10,861.7
−14,136.9	−20,589.8	−16,602.8	−20,411.0	−27,160.1	−19,604.4	−49,422.1	−21,606.9	−8968.3	−15,103.1
−24,708.9	−24,253.7	−28,946.7	−21,676.5	−22,617.6	−6704.7	−39,811.7	−7455.7	−8912.5	−32,896.1

## Appendix C

The original experiment position coordinates are shown in Table A5, Table A6 and Table A7. The experiments were performed with no compensation for the movement commands.

**Table A5:** Experiment data when moving along the path with the optimal precision. The movement commands have not been compensated (mm).

Sequence		1	2	3	4	5	6	7	8	9	10	11	12	13	14	15
1	x	0.0	0.0	0.0	0.0	0.0	0.0	0.0	0.0	0.0	0.0	0.0	0.0	0.0	0.0	0.0
	y	1000.0	1000.0	1000.0	1000.0	1000.0	1000.0	1000.0	1000.0	1000.0	1000.0	1000.0	1000.0	1000.0	1000.0	1000.0
2	x	30.8	29.6	27.9	29.7	27.8	28.9	29.0	32.2	27.1	31.1	24.3	29.2	30.9	29.3	25.1
	y	756.5	755.8	755.9	757.4	755.9	756.6	756.5	757.1	755.6	756.7	755.5	756.0	755.7	756.3	755.9
3	x	111.7	111.9	103.3	105.7	102.2	104.5	105.2	110.7	99.4	111.3	92.7	104.9	108.8	105.5	97.8
	y	427.8	427.3	425.2	426.7	425.4	425.1	426.6	426.7	424.1	427.6	423.5	425.6	424.8	424.9	424.5
4	x	171.9	171.7	161.1	164.8	160.7	163.1	164.5	170.9	157.6	170.2	149.1	163.2	167.1	164.4	155.3
	y	345.6	345.7	341.6	343.1	342.7	343.4	343.6	344.3	340.8	345.7	339.1	342.7	342.1	343.2	340.3
5	x	209.1	208.1	195.4	198.6	193.7	198.6	199.3	205.4	190.3	207.4	180.3	198.5	201.1	199.5	188.1
	y	232.9	233.2	228.6	231.0	229.0	230.4	230.0	231.6	227.4	232.4	225.5	229.3	229.1	228.6	227.5
6	x	209.3	208.8	191.6	195.5	191.6	193.9	196.7	203.6	186.8	206.0	172.7	197.5	196.1	196.7	182.6
	y	70.5	69.5	66.5	68.0	66.5	66.8	66.9	67.9	63.9	70.1	61.7	67.9	65.3	66.3	64.5
7	x	437.7	438.6	422.1	425.9	421.8	423.3	427.5	435.0	416.4	434.3	402.9	427.6	426.5	427.0	413.9
	y	89.2	89.7	79.4	84.3	80.3	80.6	81.7	84.9	75.8	88.0	70.2	82.6	79.3	83.7	75.8
8	x	658.7	659.1	642.7	646.2	641.6	643.6	646.6	655.0	635.7	656.5	622.0	648.3	646.4	649.2	631.3
	y	61.4	60.9	47.2	52.1	47.8	47.1	50.3	53.7	39.0	60.8	32.4	53.3	45.5	55.1	38.4
9	x	848.7	849.3	839.3	843.9	835.5	840.9	841.4	849.6	834.9	849.0	824.2	843.6	844.5	842.0	834.1
	y	326.1	326.7	306.6	312.6	310.5	308.1	311.6	315.6	298.6	323.4	289.7	313.9	305.6	317.3	294.9
10	x	968.9	969.8	958.5	963.2	955.3	961.2	960.9	969.5	954.4	968.7	943.4	963.1	964.3	962.6	953.8
	y	311.8	310.7	289.5	294.5	293.1	289.6	292.8	298.2	279.5	308.8	269.1	297.5	287.0	299.3	273.5
11	x	896.8	894.4	886.0	893.2	882.6	889.7	889.1	898.4	885.6	894.5	876.6	891.4	892.7	891.6	886.5
	y	454.1	452.9	433.5	438.5	437.0	432.8	437.6	441.8	422.9	450.9	415.0	441.2	429.4	443.9	418.9
12	x	918.3	916.6	913.2	920.2	908.8	917.1	917.4	922.9	911.9	916.9	909.6	915.9	917.7	919.9	919.4
	y	695.5	695.8	674.0	679.3	678.6	674.1	678.9	683.5	663.5	692.6	655.7	683.1	671.4	685.7	657.9
13	x	672.4	670.6	667.6	673.9	659.7	670.8	669.5	677.0	665.2	672.0	662.8	670.0	670.0	672.7	672.3
	y	656.8	653.8	643.1	646.7	644.9	641.7	642.1	648.6	632.0	651.5	626.8	648.5	635.9	651.6	627.5
14	x	593.9	591.3	590.0	596.5	581.7	594.3	592.5	598.2	586.9	591.5	585.6	592.1	592.4	595.0	594.4
	y	738.5	736.1	727.1	729.2	727.3	724.0	725.6	730.0	715.0	733.0	709.8	730.5	716.4	734.3	710.8
15	x	503.2	500.5	498.8	504.8	491.2	502.5	501.4	505.7	496.2	501.1	494.0	500.7	499.4	503.6	502.3
	y	763.2	759.4	753.6	753.4	752.8	750.6	748.3	754.6	741.3	756.7	736.6	756.4	740.2	758.2	736.1
16	x	408.0	402.1	406.3	414.1	400.0	411.3	404.4	411.6	408.8	403.9	408.7	410.1	406.2	408.6	414.4
	y	992.6	987.6	983.3	984.7	983.9	981.7	978.1	984.1	973.5	986.9	969.0	988.0	971.4	988.8	967.9
17	x	337.1	333.5	336.6	344.0	330.1	342.7	336.2	342.4	337.2	335.0	336.4	341.2	337.2	339.5	345.7
	y	917.9	912.1	909.4	912.7	909.7	908.9	901.7	910.9	903.0	911.2	898.9	913.2	896.9	913.0	896.1
18	x	339.0	337.9	337.7	343.1	327.6	340.5	338.2	346.1	334.1	336.7	329.8	338.1	339.3	342.0	342.6
	y	704.5	699.8	694.6	698.4	695.7	694.6	688.8	696.9	688.4	695.9	685.1	699.0	683.3	700.1	681.2
19	x	302.9	301.6	300.6	305.1	289.6	302.9	301.4	309.0	295.5	300.9	289.8	299.8	303.9	304.2	305.7
	y	649.0	642.5	639.1	643.0	639.1	638.8	632.1	639.7	633.8	640.2	630.1	644.2	626.7	643.2	625.0
20	x	283.4	285.9	281.7	285.0	270.2	282.6	284.3	292.4	273.5	281.1	267.7	279.0	286.4	287.1	284.8
	y	527.9	522.6	518.4	522.4	520.2	519.8	512.3	519.6	513.6	519.1	511.1	523.9	506.4	522.8	506.3
21	x	382.2	382.5	380.1	384.7	369.3	381.8	382.9	391.7	374.1	379.3	367.6	377.7	384.2	384.6	384.9
	y	570.0	564.3	560.1	563.1	558.0	561.9	554.4	562.3	552.4	560.5	549.5	562.8	549.6	564.3	544.7
22	x	938.2	947.0	926.5	924.2	906.6	929.1	935.9	950.7	914.1	926.9	901.8	913.0	945.1	938.5	924.8
	y	−19.8	−19.0	−39.2	−44.6	−49.3	−37.2	−38.7	−25.9	−52.4	−37.0	−61.6	−46.6	−36.2	−28.0	−59.7

**Table A6:** Experiment data when moving along the path with the least rotation. The movement commands have not been compensated (mm).

Sequence		1	2	3	4	5	6	7	8	9	10	11	12	13	14	15
1	x	0.0	0.0	0.0	0.0	0.0	0.0	0.0	0.0	0.0	0.0	0.0	0.0	0.0	0.0	0.0
	y	1000.0	1000.0	1000.0	1000.0	1000.0	1000.0	1000.0	1000.0	1000.0	1000.0	1000.0	1000.0	1000.0	1000.0	1000.0
2	x	23.9	20.2	24.0	29.6	29.3	29.5	26.6	25.9	30.4	24.5	30.4	27.8	24.9	24.2	26.2
	y	757.5	755.0	755.8	756.4	756.6	756.4	755.2	757.8	755.3	756.2	755.8	756.8	756.0	756.8	756.0
3	x	96.3	86.8	94.6	108.3	111.1	109.5	100.2	101.6	109.5	96.1	108.4	106.1	96.8	96.1	98.0
	y	425.6	423.5	423.9	425.9	427.7	427.3	424.5	428.5	427.8	425.9	426.6	426.6	424.6	426.0	426.2
4	x	153.0	143.0	152.8	167.7	170.9	168.1	158.1	159.5	167.1	153.4	166.7	164.5	154.2	153.0	155.8
	y	342.2	338.1	338.4	343.7	346.5	345.0	340.8	344.9	345.7	341.8	343.7	345.5	341.1	342.0	342.6
5	x	409.2	397.0	406.0	428.8	435.2	434.0	415.5	416.2	429.3	409.9	426.9	427.7	410.0	405.1	409.0
	y	82.0	74.9	74.3	85.6	93.8	94.2	82.8	87.1	91.6	82.5	85.9	89.9	80.8	78.1	79.0
6	x	629.2	617.6	627.6	649.7	657.9	657.9	638.0	636.7	651.7	631.8	648.6	648.4	630.1	624.8	629.2
	y	45.7	40.3	40.2	57.9	67.9	72.7	51.3	56.8	64.3	51.6	59.0	62.3	45.0	40.4	42.5
7	x	830.0	814.7	820.8	840.7	847.4	839.8	830.7	829.0	840.8	824.5	836.7	839.3	828.7	824.3	826.8
	y	304.2	301.0	305.5	324.1	336.7	343.4	313.1	320.2	328.3	311.9	323.0	326.4	304.0	299.0	301.5
8	x	879.1	863.8	865.5	883.5	891.8	882.5	877.6	876.1	886.3	873.8	883.1	885.7	878.3	874.9	878.3
	y	425.3	424.3	431.3	448.6	461.8	470.7	439.6	444.2	452.8	435.8	449.4	449.8	426.1	420.3	424.8
9	x	909.5	890.0	886.1	903.9	909.5	890.7	898.0	899.1	905.7	897.2	901.0	907.7	908.3	904.6	907.5
	y	669.1	668.4	674.6	693.4	705.5	715.5	682.1	686.8	696.3	679.1	690.9	694.6	669.6	663.0	667.3
10	x	404.9	379.9	367.4	382.1	385.9	357.3	383.1	384.3	389.4	382.8	380.8	396.2	406.8	400.3	400.6
	y	981.9	973.7	965.2	980.6	988.3	978.3	978.5	981.5	991.0	973.2	976.8	997.0	987.5	975.3	979.2
11	x	334.4	311.0	301.3	316.1	320.6	295.4	317.2	316.9	323.7	314.9	317.9	327.7	336.4	329.4	332.9
	y	908.6	900.1	889.0	903.4	910.9	900.0	901.9	905.6	914.7	896.9	898.8	922.1	915.2	902.1	906.5
12	x	373.3	352.4	360.2	372.2	382.1	361.3	366.7	363.9	372.0	361.6	374.6	368.2	370.8	368.8	367.8
	y	564.0	551.9	543.1	558.1	564.9	556.1	554.7	558.7	570.0	549.9	554.5	576.3	569.5	554.7	557.8
13	x	163.9	147.6	178.9	182.3	200.8	192.2	172.5	162.5	172.7	162.8	189.3	157.5	156.3	152.9	148.1
	y	95.5	79.7	63.3	79.1	83.8	72.1	78.4	87.7	95.0	76.4	75.2	107.8	101.4	90.0	94.8
14	x	181.4	166.0	187.3	193.4	209.5	193.8	185.5	177.4	186.1	176.4	199.8	176.2	177.1	174.4	171.1
	y	256.4	241.9	226.1	241.6	247.2	234.2	242.9	249.5	257.3	238.7	239.7	269.5	263.3	250.3	255.3
15	x	283.7	264.1	273.5	285.1	295.5	273.8	283.9	273.6	282.0	273.1	286.9	281.8	284.3	283.4	279.6
	y	519.4	507.0	496.4	510.1	516.3	505.3	507.8	515.3	522.3	502.9	506.5	530.2	525.4	509.9	516.0
16	x	307.3	284.6	288.0	303.0	312.7	286.9	302.9	295.3	303.4	292.8	302.9	305.8	309.5	310.5	304.8
	y	638.5	626.4	616.0	628.5	637.3	626.6	628.1	635.3	641.3	623.2	625.6	647.1	643.2	628.8	634.9
17	x	348.3	324.4	323.3	338.6	347.7	320.1	340.3	332.8	340.8	329.7	337.7	346.3	351.5	352.0	345.0
	y	691.7	682.1	672.7	684.6	693.3	683.9	684.5	689.0	697.8	679.7	682.6	701.5	696.8	681.2	688.7
18	x	509.5	484.2	482.0	499.8	504.9	475.7	499.8	491.3	502.2	489.3	497.1	507.3	512.6	513.9	507.3
	y	736.7	729.0	727.8	736.7	751.3	743.5	734.5	737.0	747.5	730.2	738.8	744.3	740.0	721.2	728.1
19	x	597.5	574.5	572.9	588.2	595.5	568.2	590.2	581.6	591.2	578.5	586.0	595.6	600.7	602.0	595.1
	y	705.2	700.7	703.6	708.6	726.6	720.7	708.0	708.5	718.9	702.0	712.7	712.4	708.1	688.4	694.8
20	x	669.6	646.9	651.9	664.5	674.8	646.2	662.0	654.6	664.2	651.1	660.3	665.6	669.3	670.3	665.0
	y	615.5	612.9	619.3	620.8	642.4	638.6	619.9	621.2	630.5	615.6	627.4	621.7	617.8	595.9	602.8
21	x	919.9	905.6	931.2	935.6	954.8	934.6	928.6	917.1	921.1	917.0	932.3	911.1	915.4	912.0	906.3
	y	237.5	238.7	260.9	256.0	284.7	287.9	250.8	252.5	256.1	248.1	263.0	240.1	234.3	212.1	219.1
22	x	880.3	869.6	913.5	912.0	943.3	927.4	899.0	889.6	883.5	888.1	907.4	866.0	868.0	861.1	858.9
	y	−68.1	−71.4	−49.8	−52.8	−25.5	−24.0	−60.1	−57.4	−50.8	−60.9	−45.1	−67.5	−72.2	−93.7	−87.4

**Table A7:** Experiment data when moving along the path with the shortest length. The movement commands have not been compensated (mm).

Sequence		1	2	3	4	5	6	7	8	9	10	11	12	13	14	15
1	x	0.0	0.0	0.0	0.0	0.0	0.0	0.0	0.0	0.0	0.0	0.0	0.0	0.0	0.0	0.0
	y	1000.0	1000.0	1000.0	1000.0	1000.0	1000.0	1000.0	1000.0	1000.0	1000.0	1000.0	1000.0	1000.0	1000.0	1000.0
2	x	26.7	25.5	23.3	28.7	24.0	29.5	23.2	24.1	27.3	32.5	24.6	27.2	27.9	24.9	24.9
	y	755.9	755.9	755.9	755.8	755.5	756.3	755.4	756.5	757.1	756.4	756.1	756.1	756.7	755.2	756.1
3	x	297.3	299.5	295.5	299.8	294.9	299.2	296.6	297.2	299.3	300.9	297.7	297.4	299.6	296.6	295.3
	y	895.8	897.8	897.1	898.8	896.4	899.3	891.1	890.9	899.0	903.2	895.4	900.1	898.4	898.5	899.3
4	x	360.2	362.0	358.3	361.1	357.8	361.0	360.1	361.0	360.7	360.7	360.6	358.5	360.7	358.8	358.3
	y	975.9	974.4	974.0	978.1	974.2	978.0	967.9	967.4	976.2	981.9	971.7	978.4	976.0	975.8	977.9
5	x	464.1	466.8	456.4	470.1	462.2	468.5	461.3	462.4	463.6	474.1	461.5	462.1	468.4	458.5	464.0
	y	750.0	748.9	747.0	755.4	750.1	755.7	741.6	740.7	752.4	761.3	745.7	752.5	751.9	748.5	750.7
6	x	556.8	560.4	549.1	561.4	556.5	562.1	553.4	555.1	557.8	567.8	554.0	556.1	562.8	551.3	558.2
	y	729.1	729.1	723.3	737.8	730.8	735.8	720.6	720.3	732.7	745.0	724.3	733.3	732.5	726.8	732.5
7	x	636.8	642.9	628.4	642.6	639.1	644.1	634.2	634.5	640.5	651.7	635.9	637.5	643.8	634.6	637.6
	y	648.6	649.3	642.5	658.5	652.4	655.7	639.3	638.7	652.0	666.5	642.8	654.0	653.1	645.8	653.8
8	x	882.5	888.3	874.1	888.0	882.5	889.1	879.4	880.9	884.9	895.4	880.9	882.9	889.3	878.1	884.0
	y	686.4	691.7	674.7	701.4	695.5	695.6	680.0	676.9	695.6	717.4	685.5	696.4	695.6	686.7	691.9
9	x	853.9	863.5	842.2	869.5	863.1	863.7	854.6	854.7	864.3	879.5	860.4	864.0	865.8	854.4	857.6
	y	443.6	449.2	434.2	458.2	454.3	453.4	437.2	434.5	454.4	474.0	442.0	452.6	452.4	443.5	448.2
10	x	924.1	934.9	911.7	941.3	939.8	934.2	928.0	926.2	940.7	956.2	934.3	940.1	938.7	928.7	929.7
	y	299.8	304.5	291.5	316.3	312.9	309.1	295.6	291.8	312.3	334.3	301.2	312.5	310.5	301.9	306.4
11	x	804.3	814.3	792.3	821.4	819.7	813.8	807.5	805.6	819.9	835.0	813.5	817.0	817.5	806.5	808.0
	y	323.4	325.5	315.3	334.6	330.4	332.5	314.6	313.9	333.2	352.1	319.3	334.0	329.2	324.2	328.9
12	x	365.3	361.7	354.8	369.0	363.4	373.6	357.7	358.1	366.4	381.0	356.9	367.6	364.9	354.4	362.5
	y	594.6	574.7	587.5	584.9	569.9	600.4	568.6	572.3	581.0	597.5	563.5	584.7	579.9	574.5	587.3
13	x	328.5	321.0	318.0	327.4	319.1	337.0	317.7	320.5	324.3	338.1	314.0	326.3	326.3	314.0	323.6
	y	729.8	707.5	722.6	717.7	701.6	736.3	700.9	706.2	712.9	730.3	694.4	718.1	712.9	707.2	721.3
14	x	286.7	282.1	275.7	288.1	280.7	294.1	278.3	282.4	285.8	299.5	276.6	288.3	285.7	275.3	284.9
	y	674.8	647.4	664.3	662.4	644.6	681.2	645.7	650.4	653.5	672.3	636.2	657.4	655.5	647.7	662.0
15	x	261.9	264.0	252.3	267.5	265.0	270.0	257.6	258.7	269.2	282.3	258.7	269.3	265.8	256.4	262.8
	y	554.3	528.0	544.1	542.3	525.3	562.5	523.8	529.7	534.7	551.0	515.1	539.4	534.8	527.7	543.1
16	x	88.1	90.1	77.8	95.5	95.4	95.2	84.0	86.1	97.6	110.8	86.9	96.5	93.2	82.9	91.2
	y	499.5	464.0	487.4	479.1	456.0	505.5	464.3	470.6	467.2	485.0	452.2	474.1	474.8	466.4	480.7
17	x	132.6	142.0	126.9	145.4	146.4	142.2	132.9	134.3	147.3	164.3	136.4	148.3	144.2	132.5	141.6
	y	406.7	376.7	396.6	389.9	369.1	412.9	373.6	380.8	378.7	396.8	362.5	386.7	386.1	375.7	390.9
18	x	151.3	165.8	147.7	168.4	173.0	162.6	155.3	155.3	171.4	191.5	161.2	174.3	168.2	155.1	164.4
	y	289.5	262.0	282.1	275.0	254.2	298.3	259.3	264.7	263.3	283.8	248.7	270.8	272.5	260.0	276.6
19	x	125.7	151.2	128.2	152.8	160.5	139.5	137.5	135.8	157.1	177.3	146.0	157.7	149.8	135.7	144.7
	y	129.1	98.6	121.0	110.9	90.6	137.0	95.6	101.3	102.7	120.2	85.5	109.7	108.5	98.0	114.4
20	x	357.0	381.9	359.4	384.1	390.4	369.9	366.7	366.7	386.9	408.8	375.9	390.3	380.5	367.6	376.5
	y	113.0	94.4	111.5	108.4	90.7	124.4	87.3	92.2	99.0	121.2	82.8	107.3	100.8	92.7	107.3
21	x	569.2	599.3	573.4	599.7	608.7	584.5	582.2	581.0	604.9	626.1	592.9	606.7	596.3	583.6	590.5
	y	48.6	43.2	54.2	56.2	45.0	63.7	32.0	35.7	49.2	72.9	34.2	57.4	45.0	39.6	50.3
22	x	875.1	910.8	882.3	911.2	922.1	891.5	892.7	890.3	916.0	939.9	905.8	918.7	907.3	895.7	901.1
	y	−67.1	−50.4	−51.5	−41.6	−45.5	−44.4	−70.5	−69.8	−45.3	−17.1	−59.3	−36.9	−54.8	−59.4	−55.4

## Appendix D

The original experiment position coordinates are shown in Table A8, Table A9 and Table A10. The experiments were performed by compensating the movement commands.

**Table A8:** Experiment data when moving along the path with the optimal precision. The movement commands are compensated (mm).

Sequence		1	2	3	4	5	6	7	8	9	10	11	12	13	14	15
1	x	0.0	0.0	0.0	0.0	0.0	0.0	0.0	0.0	0.0	0.0	0.0	0.0	0.0	0.0	0.0
	y	1000.0	1000.0	1000.0	1000.0	1000.0	1000.0	1000.0	1000.0	1000.0	1000.0	1000.0	1000.0	1000.0	1000.0	1000.0
2	x	27.9	33.0	29.2	29.6	31.8	29.9	29.7	27.8	22.7	32.6	28.4	26.5	28.3	28.6	22.7
	y	750.6	751.8	750.0	750.9	751.6	750.5	750.5	750.9	750.1	750.8	750.2	750.5	750.1	750.6	749.4
3	x	106.2	121.4	111.0	112.0	117.9	109.9	110.1	105.8	96.8	118.8	106.1	104.2	108.1	107.3	96.0
	y	413.6	415.8	414.7	415.6	415.2	414.3	415.7	412.1	411.0	415.2	412.0	413.3	413.1	413.4	412.2
4	x	167.4	183.2	171.6	173.3	179.5	171.5	171.7	165.8	156.7	182.2	165.5	164.9	170.3	168.0	156.6
	y	330.6	332.2	331.4	332.8	331.4	330.3	331.2	326.7	326.6	332.2	327.2	328.7	329.7	329.8	328.0
5	x	204.6	222.2	210.1	212.9	217.9	208.4	209.7	201.6	190.8	218.5	202.5	201.5	206.9	205.9	191.9
	y	215.3	220.1	217.5	218.0	218.1	215.8	217.6	212.1	211.6	218.4	213.0	214.0	216.0	215.9	213.5
6	x	206.2	224.5	212.8	215.3	220.8	210.7	210.3	200.2	188.9	220.8	201.8	202.6	209.1	207.9	191.7
	y	48.8	53.4	51.7	51.8	50.8	49.1	50.3	45.6	45.1	50.7	46.7	47.3	48.4	49.4	45.9
7	x	441.4	458.0	446.4	449.8	454.5	443.6	445.0	435.8	424.3	454.4	436.5	437.4	443.4	442.5	427.2
	y	74.0	78.5	77.1	77.3	78.8	74.5	75.1	66.8	64.8	75.8	65.7	71.4	70.5	72.0	67.1
8	x	668.2	684.5	672.5	675.9	681.5	670.1	671.3	661.0	650.0	681.5	662.6	663.2	670.3	668.6	651.9
	y	51.8	57.5	53.9	56.3	58.5	53.1	52.1	40.3	37.2	57.1	37.2	47.9	47.7	46.4	40.5
9	x	852.8	870.3	857.2	858.4	865.3	854.0	855.9	851.6	842.7	863.4	855.0	849.8	856.4	856.4	843.3
	y	329.2	335.6	331.7	333.8	336.6	331.4	329.9	315.1	308.9	337.3	310.4	324.0	325.9	324.0	312.3
10	x	977.9	994.5	981.2	982.9	988.8	976.9	980.3	975.2	966.5	988.2	979.0	974.1	980.8	980.1	967.4
	y	318.0	324.8	323.0	324.4	325.8	322.2	319.6	300.6	295.7	328.2	297.0	314.5	312.8	312.7	299.6
11	x	896.5	912.1	896.9	900.5	904.0	892.7	898.1	893.5	888.6	905.2	898.8	890.6	898.8	898.6	888.8
	y	460.4	465.9	463.6	464.8	467.3	463.5	460.5	442.6	439.9	468.2	439.6	456.1	455.5	454.9	442.0
12	x	907.9	918.8	903.0	906.2	910.1	899.5	905.5	909.5	904.7	910.7	913.0	898.1	906.5	910.6	902.3
	y	708.6	714.5	711.7	712.9	717.1	711.0	708.2	693.1	687.7	716.1	687.7	703.2	703.8	703.1	689.2
13	x	660.0	669.9	653.7	658.7	663.1	651.1	658.9	659.8	653.8	663.9	660.8	651.0	658.7	661.4	653.4
	y	655.6	660.0	653.5	657.5	658.4	654.3	651.5	644.9	638.8	657.6	637.2	646.0	648.1	650.2	639.6
14	x	573.1	583.8	567.2	570.8	576.6	563.3	571.1	576.9	568.6	574.6	576.0	562.8	570.7	575.8	566.3
	y	733.4	737.7	729.8	733.7	735.2	729.6	728.5	724.1	717.0	733.1	715.5	721.1	725.1	727.6	717.6
15	x	477.7	489.3	471.0	475.2	480.2	467.6	476.3	480.7	474.1	479.1	480.1	467.6	475.8	478.9	471.2
	y	750.1	755.5	747.0	749.5	750.1	747.1	744.8	742.1	738.5	749.4	733.1	736.6	740.2	744.9	736.3
16	x	361.8	377.0	355.0	357.0	362.9	349.0	357.3	370.3	363.9	361.7	369.8	348.3	356.9	364.4	358.6
	y	974.2	981.8	972.9	974.3	975.5	972.4	969.5	969.7	966.7	972.2	961.3	960.0	963.4	971.1	963.1
17	x	298.8	313.4	292.9	295.1	302.8	287.3	295.0	305.9	298.6	298.3	305.1	286.8	293.9	301.2	293.3
	y	890.8	901.4	889.8	891.0	893.9	889.2	886.1	890.6	886.4	888.9	882.1	875.1	880.8	889.2	881.5
18	x	322.4	337.2	313.5	321.2	328.5	310.9	319.8	323.4	313.6	325.5	320.6	311.0	320.3	320.1	312.8
	y	674.8	682.2	671.3	674.2	674.9	672.1	668.8	671.2	669.4	672.0	662.5	659.4	663.4	670.7	664.5
19	x	291.8	305.4	281.6	291.2	297.2	280.8	291.3	289.6	280.9	294.6	286.4	280.6	289.1	287.9	281.0
	y	613.1	621.1	611.1	611.9	613.0	610.8	606.1	611.7	608.7	609.1	603.5	597.2	601.0	610.0	604.0
20	x	287.8	299.6	276.3	287.0	294.1	275.9	285.9	282.3	271.3	289.8	276.9	277.3	284.7	281.5	273.0
	y	489.8	497.1	486.5	488.2	488.4	486.9	481.8	486.8	486.2	486.1	479.5	474.0	477.2	485.7	480.2
21	x	383.1	394.6	373.1	381.8	387.9	370.9	380.5	378.7	367.4	385.1	374.8	371.9	378.4	376.5	368.6
	y	543.1	550.7	538.5	543.5	544.6	540.5	536.4	536.3	534.9	540.1	528.7	528.2	531.3	538.1	532.4
22	x	1026.6	1028.8	1003.5	1027.2	1029.2	1009.0	1024.2	997.1	984.5	1025.8	989.6	1015.6	1015.0	1003.0	995.5
	y	19.1	16.4	−0.3	23.0	19.0	10.6	12.2	−15.2	−20.5	15.7	−30.3	4.8	0.3	−5.6	−10.0

**Table A9:** Experiment data when moving along the path with the least rotation. The movement commands are compensated (mm).

Sequence		1	2	3	4	5	6	7	8	9	10	11	12	13
1	x	0.0	0.0	0.0	0.0	0.0	0.0	0.0	0.0	0.0	0.0	0.0	0.0	0.0
	y	1000.0	1000.0	1000.0	1000.0	1000.0	1000.0	1000.0	1000.0	1000.0	1000.0	1000.0	1000.0	1000.0
2	x	28.9	30.5	31.4	33.1	31.9	25.3	32.2	30.2	32.6	32.7	26.1	29.4	30.6
	y	751.2	752.1	751.7	752.2	752.5	751.2	751.2	752.2	752.2	751.3	751.6	751.2	752.3
3	x	106.9	110.4	113.3	121.5	117.2	100.5	119.0	109.7	118.9	117.4	100.8	112.8	112.7
	y	414.7	417.2	416.8	419.2	418.9	415.6	416.7	418.2	417.6	417.1	415.7	415.9	417.0
4	x	166.5	170.6	174.7	184.5	178.6	160.0	181.0	169.8	180.5	179.4	161.2	174.9	174.7
	y	330.9	333.9	335.3	337.6	335.3	331.6	335.8	335.0	335.8	333.7	330.4	334.4	333.3
5	x	434.7	442.7	447.0	458.9	453.9	427.5	456.7	443.5	453.6	452.2	429.9	447.9	445.6
	y	72.4	78.9	79.5	86.7	84.2	72.8	84.9	81.0	83.0	80.4	71.3	81.9	78.5
6	x	661.4	669.2	673.3	686.2	680.5	654.1	683.0	668.6	680.7	679.6	654.5	674.4	673.0
	y	49.0	56.8	59.0	68.0	64.7	47.2	67.6	59.4	63.8	58.8	45.1	61.2	56.2
7	x	847.4	855.4	857.6	866.2	864.8	841.7	861.5	854.2	861.5	863.6	843.2	856.8	857.3
	y	324.5	332.9	336.3	348.4	343.1	322.1	348.7	335.7	343.3	336.3	320.7	338.9	333.5
8	x	890.5	897.9	899.8	906.0	904.1	883.8	899.0	894.6	900.7	904.9	885.7	897.0	897.3
	y	452.6	461.2	465.2	477.9	472.1	450.2	478.0	465.3	474.4	464.0	448.4	468.2	461.7
9	x	897.0	904.1	907.9	912.8	908.4	894.4	901.0	901.5	907.4	912.3	895.6	903.4	905.8
	y	701.4	710.6	715.8	727.4	721.0	700.5	727.6	713.8	722.9	713.5	697.5	717.7	710.6
10	x	352.0	362.0	364.9	363.0	360.9	353.9	350.7	353.9	358.7	366.7	351.9	358.2	363.6
	y	963.5	976.9	983.1	981.9	975.5	970.4	977.5	972.8	979.9	971.8	965.6	978.6	975.9
11	x	290.4	299.8	301.8	303.1	300.7	290.7	291.1	293.0	298.3	304.2	289.3	297.0	300.7
	y	881.5	895.2	901.6	896.6	891.1	889.6	892.9	889.4	897.7	888.1	884.5	895.5	893.6
12	x	371.5	381.5	376.6	388.1	386.5	365.1	376.1	376.7	379.2	385.6	367.7	378.7	380.1
	y	537.2	551.0	554.3	551.3	546.8	542.0	548.4	544.2	551.9	541.8	538.0	550.3	547.5
13	x	222.7	237.9	222.2	243.0	244.1	209.6	233.5	233.7	227.4	236.9	219.3	232.8	229.3
	y	35.2	46.1	52.4	49.0	42.6	41.7	44.2	39.8	50.5	39.3	35.7	47.5	46.1
14	x	217.6	231.0	220.5	235.4	237.6	208.0	227.7	228.3	222.8	230.2	214.4	227.4	225.0
	y	201.7	212.5	219.1	216.1	208.1	209.2	212.5	206.9	216.5	205.6	200.2	214.3	212.4
15	x	281.4	287.6	288.2	298.2	298.3	275.0	288.4	288.5	289.8	293.4	277.5	291.7	291.3
	y	481.1	493.1	498.5	497.6	488.9	488.5	493.6	488.1	496.0	486.4	480.8	493.5	491.8
16	x	285.4	290.5	294.6	302.6	301.2	279.6	292.9	292.6	295.9	297.3	280.5	297.3	296.5
	y	604.5	616.5	621.0	619.9	612.1	611.5	616.4	611.4	619.0	610.1	603.0	616.1	615.1
17	x	314.4	318.4	326.5	333.4	330.4	310.7	323.6	321.3	327.4	328.3	310.2	328.6	327.6
	y	665.2	679.1	681.5	680.4	674.2	673.9	677.3	672.6	679.8	671.2	665.3	677.9	675.2
18	x	467.7	470.9	483.1	488.7	484.2	467.1	478.0	475.8	483.7	483.9	465.5	483.7	481.6
	y	737.8	756.6	753.1	754.1	749.7	744.3	751.5	747.0	750.5	743.0	739.4	749.5	748.4
19	x	562.2	565.0	575.4	581.8	577.2	559.8	571.1	569.9	575.9	576.9	559.4	577.8	575.8
	y	723.4	744.2	737.1	739.5	736.3	728.3	736.8	731.9	732.1	727.1	724.4	734.3	733.6
20	x	651.2	653.5	660.9	670.6	666.1	644.3	658.9	657.0	661.7	664.9	645.3	664.3	662.9
	y	646.6	670.5	658.7	664.3	661.1	650.4	661.2	655.3	654.3	650.9	647.2	657.5	656.6
21	x	977.2	989.1	980.3	997.3	996.9	963.9	986.3	981.2	981.7	989.2	968.4	987.2	987.6
	y	318.3	350.2	323.4	336.3	339.4	314.0	333.3	325.3	319.1	321.1	314.7	325.3	325.8
22	x	1000.4	1021.6	998.3	1023.9	1028.9	981.5	1011.1	1006.2	1002.8	1014.5	990.2	1009.2	1011.8
	y	2.1	35.2	7.2	19.8	23.1	−1.0	18.5	9.2	2.9	5.0	−1.4	9.3	9.6

**Table A10:** Experiment data when moving along the path with the shortest length. The movement commands are compensated (mm).

Sequence		1	2	3	4	5	6	7	8	9	10	11	12	13	14	15
1	x	0.0	0.0	0.0	0.0	0.0	0.0	0.0	0.0	0.0	0.0	0.0	0.0	0.0	0.0	0.0
	y	1000.0	1000.0	1000.0	1000.0	1000.0	1000.0	1000.0	1000.0	1000.0	1000.0	1000.0	1000.0	1000.0	1000.0	1000.0
2	x	32.3	30.4	31.4	29.9	25.1	24.0	27.2	27.8	27.8	26.3	29.5	30.3	27.0	25.7	29.7
	y	752.6	751.0	752.0	750.9	749.8	751.2	752.2	750.4	750.7	751.0	750.8	750.8	750.4	751.4	751.0
3	x	304.6	303.5	304.6	303.0	301.6	301.8	304.2	303.7	303.2	301.1	304.0	302.7	304.4	301.1	303.9
	y	905.3	903.7	902.1	901.7	893.2	892.2	896.8	896.6	896.7	898.9	901.5	903.2	893.9	897.3	900.7
4	x	364.0	364.4	365.2	364.3	365.3	365.5	366.5	364.8	365.7	363.9	364.8	362.5	368.1	364.1	365.3
	y	988.0	986.2	983.7	983.4	972.4	972.4	976.4	978.8	978.1	979.6	982.3	985.1	973.8	977.0	983.5
5	x	482.4	479.2	479.2	482.5	473.0	471.8	474.2	479.4	477.7	476.8	479.2	479.7	474.1	474.3	475.9
	y	764.7	761.4	757.1	758.5	744.7	744.0	748.6	752.5	752.9	754.2	758.8	762.0	745.3	750.7	756.5
6	x	579.3	574.9	574.7	579.9	568.3	566.8	569.0	575.8	573.9	573.3	575.7	576.2	568.9	570.3	571.3
	y	748.7	744.3	740.1	743.8	724.0	722.6	729.4	734.6	735.0	738.6	741.5	746.8	724.8	732.4	737.9
7	x	668.3	662.2	659.2	668.5	653.7	653.0	654.4	663.6	661.7	660.0	662.9	664.8	656.8	656.3	657.7
	y	672.3	666.7	660.4	667.1	644.2	642.1	651.0	656.3	657.9	661.0	664.9	669.9	646.3	653.8	661.2
8	x	916.4	911.3	908.9	915.8	903.8	902.7	905.1	910.3	910.6	910.3	912.2	913.0	905.7	906.2	907.9
	y	730.2	724.6	707.6	723.8	689.5	689.4	698.6	708.5	713.8	713.5	719.4	727.9	693.7	704.8	711.2
9	x	912.9	904.5	890.6	908.6	888.0	886.3	889.4	899.1	897.4	897.5	903.6	906.2	889.4	893.7	896.5
	y	482.9	476.2	460.7	474.4	441.7	440.3	449.9	460.7	465.7	464.2	472.2	480.1	446.2	456.1	463.3
10	x	1001.1	990.1	970.0	994.6	968.2	967.0	968.9	984.2	983.8	981.5	989.2	992.7	969.9	977.7	977.9
	y	345.2	337.1	316.6	335.9	299.5	298.2	306.6	320.4	324.8	324.4	332.9	342.0	301.9	316.0	322.5
11	x	876.4	864.8	846.9	869.7	844.6	842.8	845.1	860.0	859.6	858.3	866.6	868.8	847.6	853.6	852.5
	y	352.7	349.1	330.3	344.8	315.1	315.3	324.7	334.0	336.6	337.9	343.5	351.7	318.9	329.5	336.1
12	x	391.5	382.7	373.0	384.9	374.3	370.4	373.8	379.7	377.6	377.8	382.3	381.3	373.7	373.1	377.7
	y	561.0	562.9	566.1	553.0	552.4	552.3	564.1	555.3	554.4	556.9	554.0	557.5	553.5	548.9	566.3
13	x	334.6	327.5	322.8	326.7	325.5	322.2	325.7	325.5	322.5	322.4	325.8	324.1	324.1	317.9	327.7
	y	689.8	692.5	698.4	681.1	684.0	684.7	698.0	685.7	683.8	686.2	683.9	686.3	687.0	679.1	700.1
14	x	300.3	294.5	286.5	294.7	288.3	282.9	287.2	292.0	288.4	287.8	291.0	291.0	287.3	282.3	293.2
	y	627.9	630.3	637.8	617.2	626.4	625.1	636.9	623.4	623.2	624.3	621.7	622.4	626.9	616.8	639.4
15	x	297.8	286.3	276.3	291.6	275.3	270.7	274.6	285.4	283.3	283.0	284.6	287.3	276.1	275.6	281.1
	y	503.1	506.4	515.3	492.7	501.2	501.4	514.4	500.5	499.1	500.6	498.2	500.6	503.9	492.9	515.4
16	x	132.0	118.9	105.6	125.2	103.3	99.8	102.5	118.6	116.8	116.4	116.8	121.3	105.1	107.5	112.1
	y	414.8	423.2	435.9	405.9	427.9	422.8	438.0	415.8	412.6	413.7	411.7	411.5	425.6	408.7	435.3
17	x	197.8	180.1	164.8	189.3	161.1	156.5	160.1	181.5	178.6	177.4	178.6	184.9	163.8	167.4	170.5
	y	334.1	337.8	348.6	322.7	341.5	335.7	352.2	330.6	329.1	329.5	329.3	328.9	339.8	323.2	349.4
18	x	241.0	217.8	199.2	228.8	193.0	190.5	193.3	220.0	218.0	217.2	217.3	225.7	197.7	204.2	207.4
	y	222.7	224.3	234.9	210.8	226.9	221.0	235.7	216.7	215.7	215.7	215.5	215.9	225.0	209.3	235.5
19	x	251.2	220.0	198.5	233.5	186.6	186.8	189.9	222.9	224.8	222.4	222.2	231.4	196.4	208.2	208.0
	y	54.8	56.9	67.7	44.2	61.6	54.5	68.8	50.5	50.2	50.0	49.7	48.5	58.6	43.0	68.4
20	x	484.1	453.4	432.9	467.0	421.2	420.3	426.1	457.7	457.6	454.9	457.2	466.1	430.6	442.2	440.3
	y	91.2	81.2	88.5	72.0	73.5	71.6	85.1	80.0	80.6	77.0	78.3	80.7	77.8	69.8	90.6
21	x	710.4	680.7	659.1	693.4	645.2	644.6	651.3	684.4	684.6	681.5	682.8	692.8	656.5	668.1	667.9
	y	78.4	57.6	60.0	52.5	39.0	41.9	55.0	59.8	64.6	59.9	58.6	64.5	49.8	48.1	64.7
22	x	1042.3	1009.3	985.5	1022.4	971.3	973.1	978.4	1013.1	1014.7	1013.6	1013.3	1023.7	984.8	998.3	995.8
	y	40.7	3.8	−2.0	6.1	−32.6	−21.9	−7.2	11.5	24.9	16.6	8.4	22.5	−8.5	−1.7	6.6
